# HO-1 inhibits preadipocyte proliferation and differentiation at the onset of obesity via ROS dependent activation of Akt2

**DOI:** 10.1038/srep40881

**Published:** 2017-01-19

**Authors:** Gabriel Wagner, Josefine Lindroos-Christensen, Elisa Einwallner, Julia Husa, Thea-Christin Zapf, Katharina Lipp, Sabine Rauscher, Marion Gröger, Andreas Spittler, Robert Loewe, Florian Gruber, J. Catharina Duvigneau, Thomas Mohr, Hedwig Sutterlüty-Fall, Florian Klinglmüller, Gerhard Prager, Berthold Huppertz, Jeanho Yun, Oswald Wagner, Harald Esterbauer, Martin Bilban

**Affiliations:** 1 Department of Laboratory Medicine, Medical University of Vienna, 1090 Vienna, Austria; 2 Department of Dermatology, Medical University of Vienna, 1090 Vienna, Austria; 3 Core Facilities, Medical University of Vienna, 1090 Vienna, Austria; 4 Christian Doppler Laboratory for Biotechnology of Skin Aging, Vienna, Austria; 5 University of Veterinary Medicine Vienna, 1210 Vienna, Austria; 6 Institute of Cancer Research, Department of Medicine I, Comprehensive Cancer Center, Medical University of Vienna, 1090 Vienna, Austria; 7 Center for Medical Statistics, Informatics, and Intelligent Systems, Medical University of Vienna, 1090 Vienna, Austria; 8 Department of Surgery, Division of Plastic and Reconstructive Surgery, Medical University of Vienna, 1090 Vienna, Austria; 9 Institute of Cell Biology, Histology and Embryology, Medical University of Graz, 8010 Graz, Austria; 10 College of Medicine, Dong-A University, 49201 Busan, Republic of South Korea

## Abstract

Excessive accumulation of white adipose tissue (WAT) is a hallmark of obesity. The expansion of WAT in obesity involves proliferation and differentiation of adipose precursors, however, the underlying molecular mechanisms remain unclear. Here, we used an unbiased transcriptomics approach to identify the earliest molecular underpinnings occuring in adipose precursors following a brief HFD in mice. Our analysis identifies Heme Oxygenase-1 (HO-1) as strongly and selectively being upregulated in the adipose precursor fraction of WAT, upon high-fat diet (HFD) feeding. Specific deletion of HO-1 in adipose precursors of Hmox1^fl/fl^Pdgfra^Cre^ mice enhanced HFD-dependent visceral adipose precursor proliferation and differentiation. Mechanistically, HO-1 reduces HFD-induced AKT2 phosphorylation via ROS thresholding in mitochondria to reduce visceral adipose precursor proliferation. HO-1 influences adipogenesis in a cell-autonomous way by regulating events early in adipogenesis, during the process of mitotic clonal expansion, upstream of Cebpα and PPARγ. Similar effects on human preadipocyte proliferation and differentiation *in vitro* were observed upon modulation of HO-1 expression. This collectively renders HO-1 as an essential factor linking extrinsic factors (HFD) with inhibition of specific downstream molecular mediators (ROS & AKT2), resulting in diminished adipogenesis that may contribute to hyperplastic adipose tissue expansion.

White adipose tissue (WAT) has a remarkable capacity to expand or remodel in order to meet the energy demands of the organism. In the face of caloric excess, WAT expands through the enlargement of existing white adipocytes (hypertrophy) as well as by recruitment of new fat cells (hyperplasia)^1^,^2^,^3^. Visceral adipocyte hypertrophy is detrimental for metabolic health in humans/mice^4^,^5^,^6^,^7^. However, increased fat tissue expansion resulting from adipocyte hyperplasia produces less pronounced impairments in glucose tolerance than a similar magnitude of obesity resulting from adipocyte hypertrophy^8^. Accordingly, adequate adipogenesis throughout the process of adipose expansion is a necessity to maintain metabolic homeostasis in “metabolically healthy obese humans/mice”, whereas excessive hypertrophy in the absence of the generation of new, metabolically healthy adipocytes is associated with the pathophysiology of obesity-related disease, such as diabetes and cardiovascular disease^3^,^9^,^10^,^11^,^12^. In obese humans, hyperplastic adipose tissue (many small adipocytes) is associated with better glucose, insulin and lipid profiles compared with adipose hypertrophy (i.e. few large adipocytes)^13^. In addition, there is a decreased preadipocyte frequency in visceral adipose tissue from type 2 diabetes mellitus subjects^14^. Thus, understanding the molecular and cellular mechanisms that regulate adipose homeostasis represents a promising strategy for identifying novel therapeutic opportunities to fight obesity-related complications.

Adipogenesis occurs in two steps: ‘commitment’ of adipose precursors (APs) to a preadipocyte fate and ‘terminal differentiation’ describing the process by which the preadipocyte acquires the characteristics of the mature adipocyte^1^,^2^. The majority of studies on adipocyte precursors and adipogenic differentiation had been performed *in vitro*, primarily using human and murine cell lines^15^,^16^. While these studies have provided valuable insights, most notably unraveling the adipogenic transcription cascade, when and where specific factors activate adipose precursors *in vivo* cannot be determined in the *in vitro* system^17^.

Lineage tracing studies have demonstrated that most adipose precursors within WAT are of non-endothelial and non-hematopoietic origin (CD31^−^ and CD45^−^, respectively) and express surface cell markers including CD34, CD29, as well as Platelet-derived growth factor receptor alpha (Pdgfra)^17^,^18^,^19^,^20^. These studies have shown that HFD-induced adipose tissue hyperplasia is restricted to visceral fat^19^,^21^,^22^,^23^. However, lineage tracing cannot reveal details about physiological signals and molecular mechanisms underlying the response of APs to various (patho-)physiological stimuli.

To reveal early molecular targets in APs following overnutrition, we fed mice a HFD for three days and analyzed the transcriptome of APs isolated from subcutaneous (sc) and visceral (vi) WAT. Interestingly, this approach revealed HO-1 being upregulated by HFD in APs, but not other cellular constituents of adipose tissue. Based on our previous work showing that HO-1 is a conserved pro-inflammatory mediator necessary for the adverse metabolic effects of obesity^24^, we here investigate the role of HO-1 on adipogenesis. In our previous work we deleted HO-1 in five metabolic tissues; interestingly, in three of these - beta-cells, adipose tissue (using *aP2-Cre*) and muscle - presence or absence of HO-1 appeared negligible after HFD feeding. Intriguingly though, liver and macrophage HO-1 knockout mice exhibit an exquisitely healthy metabolic profile, however, a role of HO-1 in adipogenesis was not addressed at this time, due to a lack of suitable Cre lines to delete HO-1 in adipose precursors. Although various *aP2-Cre* lines have been used to target adipose tissue and were the only ones available; the lack of specificity of these lines for adipocytes as well as expression of aP2 during terminal differentiation^25^ made this model unattractive for studying adipogenesis. We chose to readdress a role of HO-1 in adipogenesis, as HO-1 top-scored in our adipose precursor array screen, and genetic tools to target adipose precursors have now been characterized^19^. We conditionally deleted HO-1 in early (*Pdgfra-Cre*) as well as late (*AdipoQ-Cre*) adipogenesis to distinguish whether observed phenotypes are the result of gene function within adipocyte precursors (APs) or mature adipocytes. Deletion of HO-1 in AP cells using *Pdgfra-Cre* enhanced viAP proliferation and differentiation. Mechanistically, we demonstrate that deficiency of HO-1 in viAPs raised reactive oxygen species (ROS) levels and promoted proliferation and differentiation via increasing Akt2 signaling.

## Results

### HFD feeding targets HO-1 in adipose precursors

**Figure 1:**
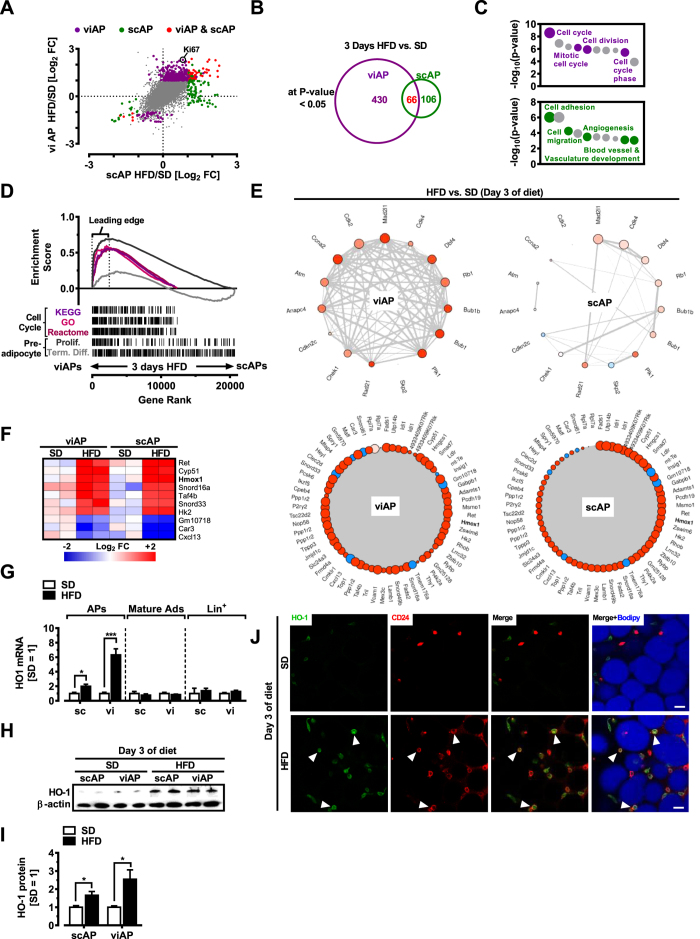
HO-1 is a target of HFD in adipose precursors. All visceral and subcutaneous APs were isolated from mice on a SD or HFD for 3 days via Sca-1 bead pull down (see ‘Experimental Procedures’). (**A**) Dot plot displaying diet-induced changes in gene expression in APs. Purple, green and red spots represent genes significantly differentially expressed greater than 2-fold (P < 0.05; n = 6 mice, pooled into two replicates per group). Grey dots indicate genes that are not statistically significantly enriched. (**B**) Venn diagram illustrating overlap between gene signatures derived from expression analysis of AP fractions. (**C**) GO analysis using DAVID^80^ of scAP and viAP molecular signatures. Notable terms are highlighted; (see also Supplemental Tables S3 + S4). P-values are derived from Fisher’s exact test and pass Benjamini-Hochberg corrected P < 0.05. (**D**) GSEA analysis of AP fractions. Analyzed gene sets include GO, KEGG and Reactome-derived cell cycle pathways as well as custom-curated genesets from a 3T3-L1 differentiation time course^31^ (see ‘Experimental Procedures’ for details). (**E**) Upper panel: Network plots for genes comprising the ‚Leading edge‘ of cell cycle from the KEGG, GO and Reactome compendium. Lower panel: Network plots for the 66 genes comprising a core HFD-associated AP gene signature. Blue node color represents downregulation and red node color represents upregulation of gene expression in APs. The node size is associated with the gene’s co-expression in the entire data set. The edge (line) thickness is linked to the gene’s connectivity (co-expression within the module). (**F**) Heatmap of top 10 genes regulated by HFD common to APs from scWAT and viWAT. Each column represents pooled cells from three mice. Blue color represents downregulation and red color represents upregulation of gene expression. (**G**) HO-1 mRNA expression in scWAT or viWAT fractions (n = 6). (**H**) Western blot of protein lysates from APs enriched from SVF of scWAT or viWAT. Each lane represents pooled cells from 2 mice. (**I**) Quantification of western blots in (H) showing HO-1 protein levels normalized to β-actin. (**J**) Whole mount immunofluorescence staining for HO-1 and CD24 in viWAT. Arrowheads indicate CD24+ cells coexpressing HO-1. Bar = 20 μm.

Short periods of consumption of a HFD (<1 wk) lead to rapid visceral-specific expansion of adipocyte precursors which is phenotypically similar to the accumulation of abdominal WAT in men^19^. However, the nutrient-associated genetic factors regulating this process *in vivo* are largely unknown. We used an unbiased transcriptomics approach to identify the earliest molecular underpinnings occuring in APs following a brief HFD in mice. Body weight as well as fat pad weight significantly increased in wildtype mice fed a HFD for three days (Figure S1A). We enriched adipose precursors by bead-purification^19^,^26^,^27^. High expression of the adipose precursor marker Pdgfra selectively in APs, paralleled by low CD68 and AdipoQ expression demonstrated successful enrichment, in addition to separation from Lin^+^ (CD31^+^CD45^+^Ter119^+^) cells and mature adipocytes (which show low Pdgfra expression) (Figure S1B). Microarray analysis was performed on AP cells lysed directly after isolation to avoid cellular phenotype alterations occuring in culture^28^. By comparing standard diet (SD)- with HFD-fed mice, microarray analysis revealed regulation of a greater number of genes in visceral APs (viAP) as compared to subcutaneous APs (scAP) (Fig. 1A and B). In viAPs from HFD fed mice 496 genes were regulated, of which 443 were induced and 53 repressed more than 2.0-fold, respectively (Fig. 1B and Table S1). In scAPs from HFD fed mice 172 genes were regulated, of which 149 were induced and 23 repressed more than 2.0-fold, respectively (Fig. 1B and Table S2). Of note, we could verify previous findings of several genes that are differentially expressed between sc and vi APs (in SD fed mice)^29^ including Tbx15, Sfrp2, Hoxc10, Ccl8, Mmp3 and others.

The HFD fed mice gene-signature in viAPs was characterized by significant enrichment of cell cycle/cell proliferation pathways, based on (i) viAP Ki67 gene upregulation (Fig. 1A), (representing proliferative capacity)^19^,^30^; (ii) DAVID classification (Fig. 1C, top panel and Table S3); (iii) GSEA analysis using curated ‘Cell cycle’ pathways from Gene Ontology, Reactome as well as KEGG. GSEA-enrichment of custom-built gene sets comprising two distinct stages of *in vitro* (3T3-L1) preadipocyte differentiation^31^: Whereas the gene set ‘terminal differentiation’ as a group exhibited less remarkable regulation, the gene set ‘preadipocyte proliferation’ showed marked upregulation specifically in viAPs from HFD-fed mice (Fig. 1D). (iv) Enrichment of a gene coexpression network representing the common genes from the GSEA-‚ ‘Leading Edge’ in viAPs (Fig. 1E, upper panel, and Table S5).

HFD feeding targets genes in scAPs associated with regulation of cytoarchitecture and/or angiogenesis (Fig. 1C, lower panel and Table S4) including Thsp1^32^, Timp1^33^ and Flt-1. Of the 172 genes whose expression is regulated by more than 2-fold in scAPs, 66 of these were also changed in viAPs (Fig. 1B and Tables S1 and S2). Thus, these 66 genes define a core HFD-induced molecular signature common to both primary visceral and subcutaneous APs, shown in Fig. 1E, lower panel, and Tables S1 and S2. The gene most highly induced in our ‘HFD-signature’ was HO-1 (Fig. 1E, lower panel & 1 F marked in bold letters). Importantly, HFD failed to upregulate HO-1 in Lin^+^ cells as well as in mature adipocytes (Fig. 1G). Significant elevation of HO-1 protein levels, detected by western blotting in isolated APs from scWAT and viWAT from mice fed either SD or HFD for three days (Fig. 1H and I), confirmed our observations at mRNA level (Fig. 1G). Enzymatic digestion of WAT followed by bead-separation may induce ‘unspecific’ gene expression in adipose cells^34^ and interfere with HO-1 expression in our APs. Thus, to exclude that experimental manipulation caused HO-1 expression, we performed histological staining of HO-1 in WAT of SD versus HFD fed mice. In SD fed mice, HO-1 staining was barely detectable, however, HFD feeding for three days strongly increased HO-1 expression. Importantly, HFD-induced HO-1 expression was found in cells expressing the adipose precursor marker CD24 (Fig. 1J). Serum of mice exposed to HFD for three days contains elevated levels of non-esterified free fatty acids (NEFAs)^35^. Therefore, we assessed whether fatty acids are responsible for HO-1 induction in APs. Indeed, incubating these cells with palmitic acid resulted in strong upregulation of HO-1 mRNA in a time-dependent manner (Figure S1C), in contrast to linoleic and oleic acid (Figure S1D). Further, HO-1 upregulation was dependent on the transcription factor Nrf2, since palmitic acid failed to induce HO-1 mRNA in APs from Nrf2 knockout mice (Figure S1E). Thus, these data strongly support our notion that HO-1 is part of an early molecular mechanism determining AP activation during HFD feeding. Together, our data show that HFD feeding triggers depot-specific-, as well as depot-independent gene expression signatures in APs *in vivo*, and that viAPs express a proliferation network which is absent in scAPs. Among the top-regulated genes in APs from both fat depots we found HO-1, upregulated by HFD almost exclusively in the AP-fraction of WAT, but not Lin^+^ or mature adipocyte cells, suggesting that HO-1 may transduce HFD effects in APs *in vivo*.

### HO-1 blocks HFD-induced visceral adipose precursor proliferation

**Figure 2:**
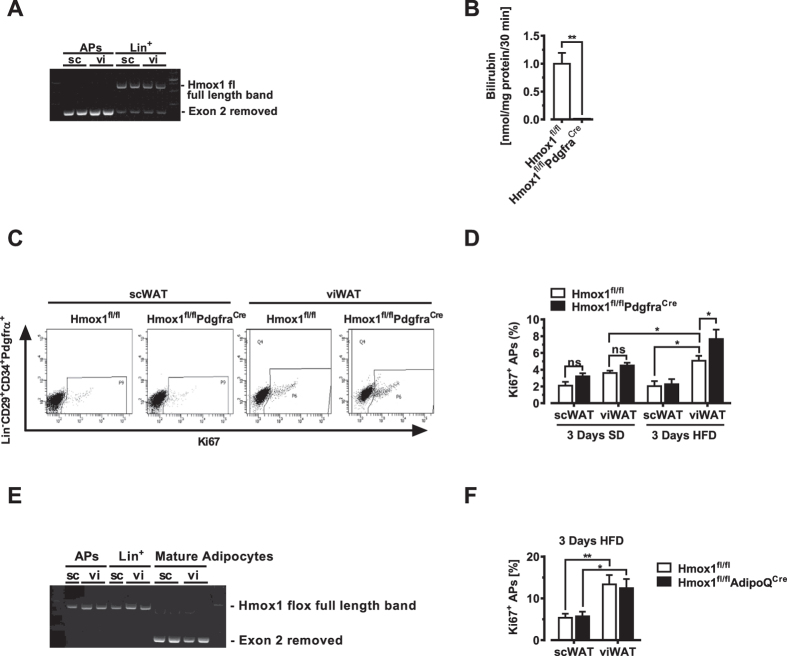
HO-1 blocks HFD-induced adipose precursor proliferation. (**A**) PCR analysis of DNA isolated from bead-purified APs and Lin^+^ cells from the viWAT and scWAT depots of Hmox1^fl/fl^Pdgfra^Cre^ mice. (**B**) HO-1 activity in APs, challenged with hemin for 16 hrs (n = 5). (**C**) Exemplary flow cytometry plots of Ki67 staining in APs obtained from the SVF of scWAT or viWAT from Hmox1^fl/fl^ and Hmox1^fl/fl^Pdgfra^Cre^ mice after feeding a HFD for 3 days. (**D**) Quantification of Ki67 in APs obtained from Hmox1^fl/fl^ and Hmox1^fl/fl^Pdgfra^Cre^ mice (n = 6). (**E**) PCR analysis of DNA isolated from AP cells from the viWAT and scWAT depots of Hmox1^fl/fl^AdipoQ^Cre^ mice. (**F**) Quantification of Ki67 in APs obtained from Hmox1^fl/fl^ and Hmox1^fl/fl^AdipoQ^Cre^ mice. n = 6 per group. Results are mean ± SEM. *p < 0.05, **p < 0.01, ***p < 0.001.

To test whether HO-1 affects the proliferation gene network in adipose precursors *in vivo*, we generated a conditional HO-1 knockout with the *Pdgfrα* promoter, which targets adipocyte precursors, but not liver or muscle^17^,^19^,^22^,^36^. We found that full excision of the HO-1 gene is achieved in APs from both scWAT and viWAT, while Lin^+^ cells still contain the full-length Hmox1 gene (Fig. 2A). Transgenic Hmox1^fl/fl^Pdgfra^Cre^ mice were born at the expected mendeleian frequency, with no observed signs of abnormality, illness or increased mortality and exhibited normal body weight at six weeks of age (Figure S2A). HO-1 levels in APs from unchallenged, naive mice are barely detectable as seen in Fig. 1G–I, so to confirm efficient HO-1 protein deletion in APs, the bead purified APs were further stimulated with the strong HO-1 inducer, hemin. Consistently, hemin treatment resulted in reduced heme oxygenase activity in APs derived from Hmox1^fl/fl^Pdgfra^Cre^ mice compared with Hmox1^fl/fl^ littermates (Fig. 2B). *In vivo*, an initial burst of AP proliferation precedes differentiation into mature adipocytes. Thus, we focused our analysis on the Lin^−^:CD29^+^:CD34^+^:Pdgfrα^+^ population of stromal cells within WAT as candidate adipose precursors and assessed their proliferation by intracellular staining for the cell proliferation antigen Ki67 (Figure S2B). On standard diet, flow cytometry analysis of APs freshly isolated from adipose SVF showed that Ki67 values were similar in APs from Hmox1^fl/fl^Pdgfra^Cre^ and Hmox1^fl/fl^ control mice. However, when these mice were challenged with a HFD for a short period of time (3 days), we found a significant increase in AP proliferation in viWAT of Hmox1^fl/fl^Pdgfra^Cre^ mice compared to HFD-fed controls (Hmox1^fl/fl^) (Fig. 2C and D, right panel), despite similar food intake (Figure S2C), whereas APs derived from scWAT, proliferation levels were similar (Fig. 2D, left panel). To eliminate the possibility that HO-1 from mature adipocytes could have an effect on AP proliferation, we generated mice in which the Hmox1 gene was selectively deleted in differentiated adipocytes (designated Hmox1^fl/fl^AdipoQ^Cre^, Fig. 2E). Consistent with its primary role in APs, deletion of HO-1 in mature adipocytes did not affect AP proliferation (Fig. 2F).

### HO-1 inhibits adipogenesis in primary adipose precursor cells isolated from mice fed a brief HFD

**Figure 3:**
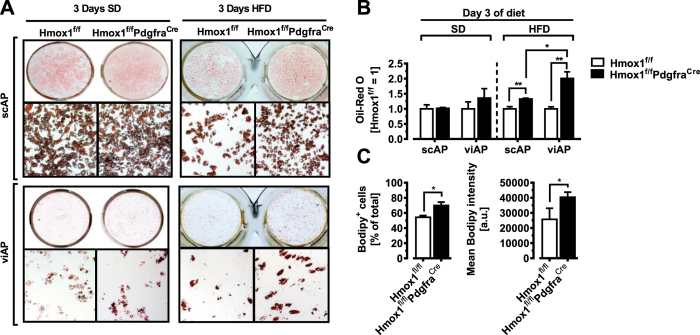
HO-1 inhibits adipogenesis in primary adipose precursor cells isolated from mice fed a brief HFD. All visceral and subcutaneous APs were isolated from mice on a SD or HFD for 3 days via Sca-1 bead pull down (see ‘Experimental Procedures’). (**A,B**) Oil-Red O staining of scAP or viAP cell cultures derived from Hmox1^fl/fl^ and Hmox1^fl/fl^Pdgfra^Cre^ mice 7 days after the induction of adipocyte differentiation (n = 3). (**C**) Flow cytometry for viAP cell cultures derived from Hmox1^fl/fl^ and Hmox1^fl/fl^Pdgfra^Cre^ mice Bodipy lipid-stained 4 days after the induction of adipocyte differentiation, measured as mean fluorescence intensities (n = 3). Results are mean ± SEM. *p < 0.05, **p < 0.01, ***p < 0.001.

Bead-purified APs from Hmox1^fl/fl^Pdgfra^Cre^ and Hmox1^fl/fl^ control mice were induced to differentiate into mature adipocytes by treatment with an adipogenic induction cocktail containing dexamethasone, IBMX, TZD and insulin. APs derived from sc- and viWAT from unchallenged naive (SD-fed) Hmox1^fl/fl^Pdgfra^Cre^ and Hmox1^fl/fl^ mice exhibited identical adipogenic capacity, as measured with Oil Red-O staining for triglycerides (Fig. 3A, left panel & Fig. 3B). An expected result as HO-1 is largely absent in APs from unchallenged naive mice, as shown in Fig. 1H. Therefore, we fed Hmox1^fl/fl^Pdgfra^Cre^ and Hmox1^fl/fl^ control mice a HFD for three days prior to AP isolation, resulting in strong HO-1 induction in APs (Fig. 1G–I). Under those obesogenic conditions, treatment with an adipogenic cocktail resulted in more efficient adipogenesis in APs isolated from HFD-fed Hmox1^fl/fl^Pdgfra^Cre^ mice as compared with APs isolated from Hmox1^fl/fl^ control mice (Fig. 3A, right panel & Fig. 3B). Noteworthy, not only the number of lipid accumulating cells, but also the intracellular lipid content was higher when viAPs were differentiated from Hmox1^fl/fl^Pdgfra^Cre^ mice as compared with Hmox1^fl/fl^ control mice (Fig. 3C).

### HO-1 inhibits early events during adipogenesis in a cell-autonomous manner

**Figure 4:**
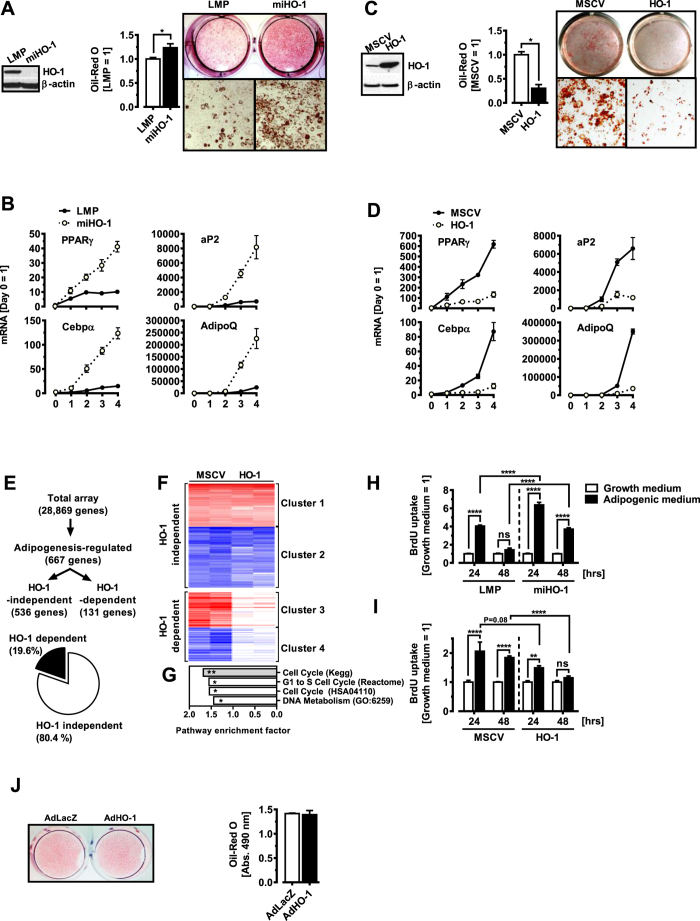
HO-1 inhibits early events during adipogenesis in a cell-autonomous manner. (**A**,**C**) Left panels: 3T3-L1 preadipocytes were transduced with (**A**) a retrovirus expressing a shRNAmir specific for HO-1 (miHO-1) or ctrl LMP vector (LMP), or with (**C**) a retrovirus expressing HO-1 or empty MSCV vector, and protein lysates were prepared for Western blotting at confluence. β-actin demonstrates equal protein loading. Middle and right panels: Oil-red O staining was performed on day 7 after induction of adipocyte differentiation. (**B**,**D**) Q-PCR analysis of adipocyte genes PPARγ, CEBPα, Adiponectin and aP2 were analyzed with Q-PCR on days 0, 1, 2, 3 and 4 after induction of adipocyte differentiation. (**E**) Microarray analysis of control (MSCV) or HO-1 overexpressing (MSCV-HO1) 3T3-L1 cells 8 hrs after addition of an adipogenic cocktail. (**F**) Heatmap of HO-1 independent (Cluster 1 +2) and HO-1 dependent (Clusters 3 + 4) genes following addition of an adipogenic cocktail (n = 2 biological replicates). (**G**) Pathway analysis of HO-1 dependent genes (Clusters 3 + 4). (**H**,**I**) BrdU incorporation into HO-1 gain- and loss of function 3T3-L1 cells 24 and 48 hrs after addition of an adipogenic medium (n = 4). (**J**) 3T3-L1 cells were induced to differentiate into mature adipocytes. 48 hrs later, cells were infected with a control (AdLacZ) or HO-1 overexpressing (AdHO-1) adenovirus and differentiation was continued for another 5 days followed by Oil red-O staining. Results are mean ± SEM. *p < 0.05, **p < 0.01, ***p < 0.001, ****p < 0.0001.

Besides HO-1 induction, HFD feeding will have ‘off target’ effects, therefore we turned to 3T3-L1 cells (which endogenously express HO-1), and generated gain- as well as loss of function cells. Cells with reduced HO-1 demonstrated enhanced adipogenic potential, including greater lipid accumulation (Fig. 4A) and increased expression of adipocyte marker genes such as PPARγ, CEBPα, FABP4 and Adiponectin (Fig. 4B). Overexpression of HO-1 in these cells markedly blocked adipogenesis, as shown by reduced Oil-red O staining of neutral lipids (Fig. 4C). Adipocyte markers were significantly decreased in HO-1-overexpressing cells (Fig. 4D), further confirming an antiadipogenic effect of HO-1. We applied microarray experiments comparing control (MSCV) versus HO-1 (MSCV-HO1) over-expressing cells treated with an adipogenic cocktail (Fig. 4E). Pathway analysis of genes affected by HO-1 expression (Fig. 4F) demonstrates an inhibitory role of HO-1 on cell cycle as well as proliferation-related events (Fig. 4G). To test whether HO-1 affects early clonal expansion, which is a prerequisite for 3T3-L1 adipocyte differentiation^37^, we performed BrdU-incorporation assays in transgenic 3T3-L1 cell lines treated with growth medium or an adipogenic medium. Treatment of control cells (LMP) with an adipogenic medium increased BrdU incorporation, an effect that was significantly enhanced in HO-1 knockdown cells (miHO1) (Fig. 4H). Accordingly, HO-1 overexpression (MSCV-HO1) resulted in significantly reduced BrdU incorporation as compared with control cells (MSCV) following treatment with an adipogenic medium (Fig. 4I). Within 48 hrs of adipogenic stimulation, growth-arrested preadipocytes re-enter the cell cycle and undergo two rounds of proliferation, a process termed ‘mitotic clonal expansion’ (MCE), ultimately leading to the upregulation of PPARγ and CEBPα^37^. While HO-1 overexpression *before* adipogenic induction reduced the accumulation of intracellular TGs as well as expression of PPARg, Cebpa and AdipoQ, (Fig. 4C,D), HO-1 overexpression *after* MCE had no effect on adipogenic gene expression on day 7 as well as TG accumulation (Fig. 4J). We conclude that HFD feeding induces HO-1 in APs and HO-1 cell-autonomously inhibits their subsequent differentiation into mature adipocytes via interfering with early adipose precursor activation (i.e. proliferation), upstream of Cebpα and PPARγ.

### HO-1 prevents HFD-induced Akt2 signaling in visceral adipose precursor cells

**Figure 5:**
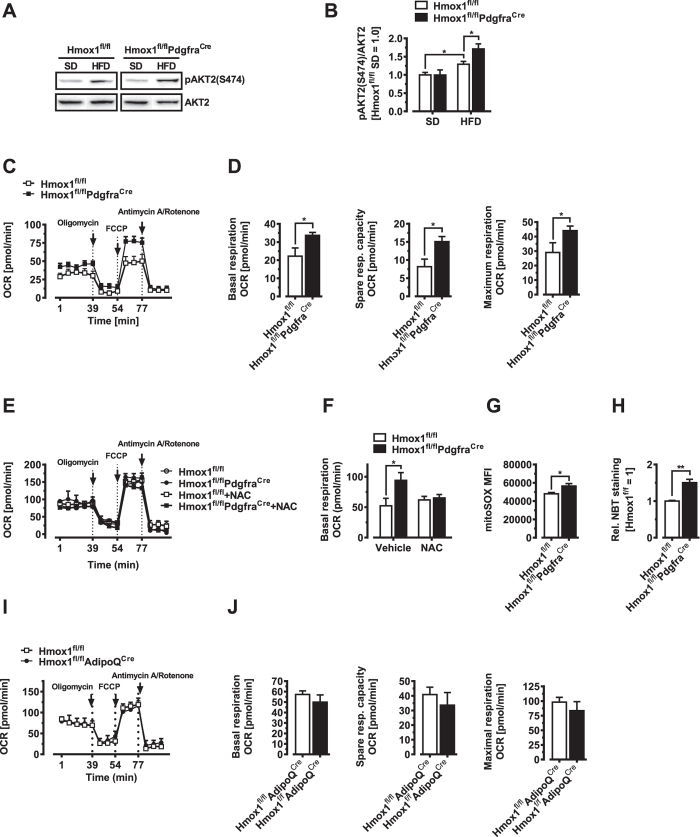
HO-1 prevents HFD-induced Akt2 signaling in visceral adipose precursor cells. (**A**) pAKT2(S474) and total AKT2 levels were measured by immunoblotting of bead purified viAPs from Hmox1^fl/fl^ and Hmox1^fl/fl^Pdgfra^Cre^ mice fed either SD or HFD for 3 days. A representative western blot is shown. The experiment was repeated at least three times. (**B**) Quantification of the pAKT2(S474)/AKT2 ratio. (**C**,**D**) Oxygen consumption rates (OCR) and mitochondrial function of viAPs derived from Hmox1^fl/fl^ and Hmox1^fl/fl^Pdgfra^Cre^ mice (n = 4). (**E**,**F**) Oxygen consumption rates (OCR) and mitochondrial function of viAPs derived from Hmox1^fl/fl^ and Hmox1^fl/fl^Pdgfra^Cre^ mice. N-acetyl-cysteine (NAC, 10 μM) was added 1 hr before measurements (n = 4). (**G**) Flow cytometric quantitation of mitoSOX-stained viAPs derived from Hmox1^fl/fl^ and Hmox1^fl/fl^Pdgfra^Cre^ mice (n = 6). (**H**) NBT reduction staining of viAPs derived from Hmox1^fl/fl^ and Hmox1^fl/fl^Pdgfra^Cre^ mice, after 48 hrs in culture (n = 4). (**I–J**) Oxygen consumption rates (OCR) and mitochondrial function of viAPs derived from Hmox1^fl/fl^ and Hmox1^fl/fl^AdipoQ^Cre^ mice (n = 4). Results are mean ± SEM. *p < 0.05, **p < 0.01, ***p < 0.001.

**Figure 6:**
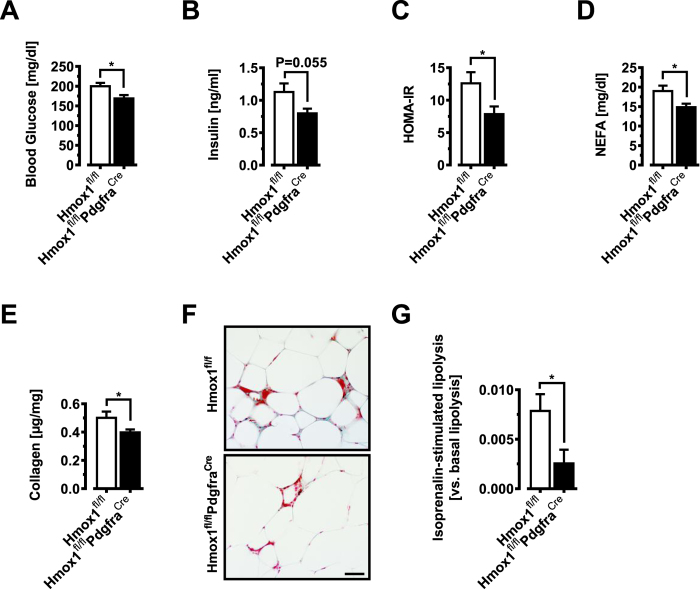
Adipose precursor HO-1 deletion affects WAT function during obesity. All mice (Hmox1^fl/fl^ and Hmox1^fl/fl^Pdgfra^Cre34^) were kept on HFD for 8 weeks. (**A–D**) Fasting circulating levels of glucose (**A**) and insulin (**B**) in obese mice. (**C**) HOMA-IR (n = 10–12). (**D**) Serum free fatty acids (NEFA) in obese mice (n = 10–12). (**E**) viWAT collagen content of Hmox1^fl/fl^ and Hmox1^fl/fl^Pdgfra^Cre^ mice (n = 6). (**F**) Trichrome staining of visceral adipose tissue of Hmox1^fl/fl^ and Hmox1^fl/fl^Pdgfra^Cre^ mice. Bar = 50 μm. (**G**) Isoprenaline stimulated lipolysis in viWAT pieces from Hmox1^fl/fl^ and Hmox1^fl/fl^Pdgfra^Cre^ mice (n = 6). Results are mean ± SEM. *p < 0.05, **p < 0.01, ***p < 0.001.

We next pondered on how HO-1 blocks AP proliferation. Phosphorylation of Akt2 has been shown to mediate the effects of HFD on AP proliferation^19^. HFD feeding for three days elevated phosphorylated AKT2 at S474 in bead-isolated, uncultured viAPs in our control (Hmox1^fl/fl^) mice, as described previously^19^; and more importantly, pAKT2 levels were higher in viAPs of Hmox1^fl/fl^Pdgfra^Cre^ mice compared to Hmox1^fl/fl^ littermate controls (Fig. 5A and B). We have previously shown that HO-1 triggers mitochondrial changes that contribute to the “pre”-programming of cellular function in macrophages and hepatocytes^24^. We therefore investigated if HO-1 exerts similar effects in APs, linking adipose precursor HO-1 with the well documented role of mitochondrial metabolism in adipogenesis^38^. Intriguingly, when assessing mitochondrial activity in naive viAPs, we found clearly elevated basal, spare as well as maximal respiratory capacity in the HO-1-deficient cells (Fig. 5C,D). Recent studies have suggested that mild ROS elevations activate mitochondrial respiration^39^. If ROS balance was altering oxidative capacity (Fig. 5C), mitochondrial function in HO-1 knockout cells should be particularly sensitive to antioxidants. In line with this idea, 1 hr preadministration of the ROS quencher N-acetyl-cysteine (NAC), reverted HO-1- deficient mitochondrial functional parameters back to control levels (Fig. 5E,F). Further, ROS levels were significantly increased in viAPs derived from Hmox1^fl/fl^Pdgfra^Cre^ mice based on elevated measures of MitoSOX fluorescence, a probe that selectively detects mitochondrial superoxide and nitroblue tetrazolium (NBT) reduction staining (Fig. 5G,H), suggesting that HO-1 regulates acute ROS thresholding of mitochondrial respiration in APs. Finally, we also tested viAPs from Hmox1^fl/fl^AdipoQ^Cre^ mice to exclude any possible influence of HO-1 from mature adipocytes. As expected loss of HO-1 in adipocytes had no effect on mitochondrial activity in APs (Fig. 5I,J). Mechanistically, our data suggest that HO-1 continuously limits activation of key signaling systems (PI3K-AKT2), controlling adipose precursor activation through an acute and ROS-dependent mechanism. Taken together, these data indicate that visceral APs activated very early after high-fat diet feeding, undergo adipogenesis more efficiently when HO-1 levels are reduced, facilitating ROS thresholding of mitochondrial respiration upstream of AKT2. We further tested if these mice exhibit any metabolic abnormalities after an extended HFD feeding period of 8 weeks. Intriguingly, fasting blood glucose and insulin as well as Homeostasis model assessment of insulin resistance (HOMA-IR) levels were lower in Hmox1^fl/fl^Pdgfra^Cre^ as compared to Hmox1^fl/fl^ control mice (Fig. 6A–C), indicating improved metabolic health in the HFD-treated Hmox1^fl/fl^Pdgfra^Cre^ mice. Serum levels of free fatty acids were significantly lower in HFD-fed Hmox1^fl/fl^Pdgfra^Cre^ mice compared to their control littermates (Fig. 6D). Irrespective of diet, Hmox1^fl/fl^Pdgfra^Cre^ and Hmox1^fl/fl^ littermates gained weight to a similar extent, and displayed similar fat pad weights (Figure S3A–D). Histological examination of scWAT and viWAT of Hmox1^fl/fl^Pdgfra^Cre^ mice revealed a trend towards higher number of small adipocytes in viWAT from Hmox1^fl/fl^Pdgfra^Cre^ mice (Figure S3E). To explain the metabolic improvements observed in Hmox1^fl/fl^Pdgfra^Cre^ mice we investigated adipose tissue fibrosis and *ex-vivo* lipolysis in viWAT. Total collagen content as well as trichrome staining demonstrated reduced tissue fibrosis in viWAT of obese HFD-fed Hmox1^fl/fl^Pdgfra^Cre^ mice compared with Hmox1^fl/fl^ control mice (Fig. 6E,F). Because serum fatty acid levels can be a reflection of adipose tissue lipolysis^40^, *ex vivo* lipolysis of viWAT was analyzed. In fact, viWAT explants from obese HFD-fed Hmox1^fl/fl^Pdgfra^Cre^ mice showed reduced isoprenaline stimulated lipolysis when compared with Hmox1^fl/fl^ control mice (Fig. 6G). Together, our data show that obese Hmox1^fl/fl^Pdgfra^Cre^ mice lacking HO-1 specifically in adipose precursors display reduced markers of metabolic disease, associated with a decrease in lipolysis and adipose tissue fibrosis.

### HO-1 impairs human adipocyte proliferation and differentiation

**Figure 7:**
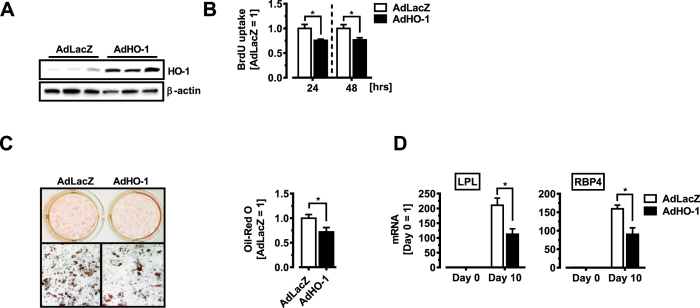
HO-1 impairs human preadipocyte proliferation and differentiation. All hASC were transduced with an Adenovirus for LacZ (AdLacZ) or HO-1 (AdHO-1) and experiments started 48 hrs later. (**A**) Western blot analysis of HO-1 protein expression in human ASC. β-actin demonstrates equal protein loading. (**B**) Cell proliferation analysis in human ASC. (**C**) Human ASC transduced with an Adenovirus for LacZ (AdLacZ) or HO-1 (AdHO-1) were induced to differentiate into mature adipocytes. Oil-red O staining was performed on day 10 after induction of adipocyte differentiation. (**D**) Q-PCR analysis of mature adipocyte genes LPL and RBP4 analyzed with Q-PCR 10 days after induction of adipocyte differentiation. Results are mean ± SEM. *p < 0.05, **p < 0.01, ***p < 0.001.

To investigate if HO-1 exerts similar effects on human adipogenesis, we used primary adipose stromal cells (hASC) isolated from lipoaspirates^41^. In order to manipulate HO-1 levels, we selected an overexpression strategy based on our observations that endogenous HO-1 levels in hASC are low. Transient HO-1 overexpression was achieved by transduction of hASC with an adenovirus carrying HO-1 expression construct (Fig. 7A)^24^. We determined if HO-1 affects hASC proliferation using an *in vitro* BrdU cell proliferation assay. Following plating of equivalent numbers of adenovirally-transduced hASC, AdLacZ-transduced cells readily incorporated BrdU 24 and 48 hrs later, an effect that was reduced when HO-1 was overexpressed (Fig. 7B). When induced to differentiate into mature adipocytes, control- (AdLacZ) transduced hASC showed prominent lipid accumulation as revealed by Oil-Red O staining. However, when hASC were instead transduced with an HO-1 adenovirus (AdHO-1), adipocyte differentiation was markedly reduced (Fig. 7C). Adipocyte markers were significantly decreased in HO-1-overexpressing cells, evaluated at day 10 of differentiation (Fig. 7D). Thus, elevated HO-1 levels act anti-proliferative and anti-adipogenic in murine and human adipose precursor cells.

## Discussion

In this study, we aimed to identify novel HFD target genes involved in the activation of adipose precursor cells, on the basis of (1) a hitherto unknown function in adipocyte biology, (2) a robust induction upon HFD feeding in APs *in vivo*, and (3) the potential to act upstream of the adipogenic master regulators Cebpα and PPARγ. Using these criteria, we identified among the top-most regulated genes, HO-1. Conditional HO-1 knockout mouse models using *Pdgfra*-Cre as well as *AdipoQ*-Cre reveal a novel role for HO-1 in pathways that govern obesogenic adipocyte proliferation and differentiation.

Although APs from different WAT depots have distinct gene signatures^29^,^42^,^43^,^44^,^45^, it is not clear if these cells also differ in their response to environmental cues, such as hormones or overfeeding. Our microarray screen using purified murine sc- and viAPs^19^,^26^,^27^ extends on these studies providing potential molecular underpinnings of hyperplastic fat expansion, some of which have a documented role in adipogenesis, mostly via facilitation of clonal expansion, including Cyclins -D1 & -D3, -E1 & -E2 as well as Cdk-2 and -4^46^,^47^,^48^. Some of the genes targeted by HFD in scAPs are (dys)regulated in obesity and/or have a documented role in regulation of extracellular matrix (ECM) architecture and angiogenesis in adipose tissue such as Tsp1^32^, Timp1^33^ or Flt1^49^. Maintaining a high degree of flexibility of the ECM allows the AT to expand in a healthy manner, without adverse metabolic consequences^1^,^6^,^50^,^51^. Further studies are needed to investigate if these genes play a role in the postulated function of APs as being sensors of overnutrition^52^,^53^.

Pdgfra^+^ APs responded to nutrient overload in WAT with proliferation as early as 24 hours following nutrient overload^17^,^18^,^21^,^52^,^53^. Biochemical data using purified APs show that HO-1 is a HFD target gene in APs from both depots, making HO-1 a candidate gene with the potential to regulate the ‘adipogenic’ response. Mice fed a HFD for three days display elevated NEFAs^35^, and we identified palmitic acid as such a HFD-elicited signal responsible for upregulation of HO-1 in APs, dependent on Nrf2. The expression of several cell cycle regulators identified in our screen were enhanced in viAPs from our AP-specific HO-1 knockout mice suggesting a direct role of HO-1 on obesogenic AP activation. Enhanced Ki67 staining, as shown by FACS analysis in Pdgfra^+^ APs from HO-1 knockout mice, further support a role of HO-1 in AP proliferation at the onset of obesity. BrdU-uptake assays in 3T3-L1 cells, engineered for HO-1 gain- and loss of function, further support an inhibitory role of HO-1 on adipogenesis-induced AP proliferation and positions HO-1 in the ‘commitment’ phase of adipogenesis. The depot-specific action of HO-1 on visceral, but not subcutaneous AP proliferation may likely be explained by the recent observations that obesogenic AP proliferation is restricted to this compartment^19^,^23^,^54^,^55^. Future studies will identify potential effects of HFD-induced HO-1 in subcutaneous APs.

In addition to enhanced AP proliferation, AP differentiation was also enhanced in HO-1 deficient APs (Fig. 3). A reduction in adipogenesis was observed solely when APs were isolated from HFD-fed mice, but not from chow fed animals, a phenomenon that may be explained by the finding that HO-1 levels are (too) low in APs from chow-fed mice (Fig. 1H). APs activated during the first week on HFD, undergo adipogenesis to form new adipocytes over a period of several weeks^19^,^23^,^43^. Remarkably, adipocyte size distribution and viWAT weight remained unaffected. Further studies utilizing longer HFD durations and examination of the development of other WAT depots such as retroperitoneal and mesenteric WAT^56^ as well as the use of alternative genetic tools to mark adipose precursors (e.g. Pdgfrb-rtTA; TRE-Cre “MuralChaser mouse”^57^) are needed. The formation of adipocytes during development can vary greatly from the route by which new adipocytes are recruited in response to overnutrition. Interestingly, both, Hmox1^fl/fl^Pdgfra^Cre^ and Hmox1^fl/fl^ control mice mice fed a standard diet display no differences in adipocyte differentiation and/or recruitment of APs. This suggests that HO-1 plays a negligible role during physiologic WAT development. The most obvious explanation for this finding may be that HO-1 is largely absent in APs from ‘unstressed’ animals (Fig. 1H). In stark contrast, HO-1 is markedly induced in stressed animals, fed a HFD for even a short period of three days, resulting in a consecutive block of adipose precursor cell proliferation and differentiation. Forced overexpression of HO-1 in 3T3-L1 and human scWAT lipoaspirates strongly support the cell-autonomous effects of HO-1 on adipogenesis. Intriguingly, HO-1 & Akt2 may be functionally linked as modulation of both, HO-1 and Akt2 signaling affects adipose precursor proliferation in the setting of overnutrition, yet dispensable for normal development of WAT. Our data showing enhanced phosphorylation of Akt2 in HO-1 deficient cells (Fig. 5A and B) would support this notion and place HO-1 upstream of Akt2. We cannot exclude that other pathways more upstream of Akt2 are also co-effected by HO-1, which needs to be evaluated by future studies, but for now we focused on Akt2 because of its critical role in AP proliferation^19^. Importantly, the anti-proliferative and anti-adipogenic effect of HO-1 was also observed in primary human preadipocytes which could potentially be important for humans, considering that fat cell numbers increase, also in humans, when overfed for a short period of time^58^. Pharmacologic HO-1 induction has provided conflicting data on the role of HO-1 in adipocyte differentiation^59^,^60^,^61^,^62^. “Off-target” effects of pharmacologic HO-1 inducers (discussed in ref. 24), and the use of bone marrow- as opposed to adipose tissue derived mesenchymal stem cells in these studies may explain the discrepancies, considering the cell-autonomous differences between WAT- and bone marrow derived ‘preadipocytes’^63^,^64^. Mechanistically, HO-1 acts upstream of Cebpα and PPARγ, as overexpression of HO-1 in 3T3-L1 cells which require MCE to trigger the terminal differentiation transcription cascade, blocks their differentiation only when overexpressed *before* MCE occurs (Fig. 4C vs J). Our data suggest a trigger-like role for HO-1 in adipogenesis by compromising mitochondria-derived ROS signaling, a scenario shared by macrophages and hepatocytes^24^. Evidence is accumulating that “low amounts” of ROS are actually fundamental signaling molecules in many cellular processes, promoting stress resistance, and that excessive ROS scavenging may have damaging side effects^65^. Our findings that elevated mitochondrial activity/ROS levels in HO-1 deficient APs display enhanced proliferation and differentiation capacities are consistent with ROS facilitating fat cell formation^38^,^66^,^67^,^68^. Here, we add HO-1 as a new upstream building block of such redox-dependent signal “preconditioning” *in vivo* and suggest a model whereby Akt2 acts as a transducer of HO-1 dependent ROS levels to support AP proliferation. This collectively renders HO-1 as an essential factor linking extrinsic factors (HFD) with specific molecular mediators, resulting in diminished adipogenesis.

Our goal was to evaluate functional effects that occur when activated APs have differentiated into mature adipocytes, a process that requires up to 7 weeks to complete^19^,^23^,^55^, before metaflammation occurs^69^. Hmox1^fl/fl^Pdgfra^Cre^ mice fed a HFD for 8 weeks displayed reduced levels of several metabolic parameters (Fig. 6A–D), despite similar fat pad weights. Several possible mechanisms may exist: (i) reduced lipolysis in viWAT examined *ex vivo* (Fig. 6G), (ii) reduced ECM deposition (Fig. 6E,F), (iii) enhanced lipid filling capacity of activated APs from Hmox1^fl/fl^Pdgfra^Cre^ mice over the 8 week HFD time course. It is well established that adipose tissue fibrosis as well as elevated FFAs cause systemic metabolic alterations^6^,^70^. Our data are in line with a previous report linking reduced lipolysis and free fatty acids with improved glucose and insulin metabolism in adipose tissue G_S_α-deficient mice, despite similar adiposity^71^. We recently demonstrated that macrophage as well as hepatocyte HO-1 is a major driver of metaflammation^24^. Our results are in line with these studies and place adipose precursor HO-1 upstream, and show that HO-1 induction in APs happens very early in a cascade leading to metabolic dysfunction. In this study, we demonstrate that HO-1 regulates the HFD-induced activation of visceral adipose tissue. In the absence of adipose precursor HO-1, proliferation of viAPs in HFD-fed mice is increased, resulting in a clear improvement of WAT function and important metabolic parameters.

## Methods

### Animals

Animal procedures were approved by the Austrian Ministry for Science and Research and the experiments were carried out in accordance with the approved guidelines and regulations. All mice used for these studies were on the C57BL/6 J genetic background. Hmox1^fl/fl^ mice contain LoxP sites flanking exon 2 of the HO-1 gene locus and were described previously^24^. Elimination of exon2 leads to a consecutive frameshift in exon 3, an early stop codon, thus resulting in a truncated peptide consisting of 9 amino acids^24^. Pdgfra^Cre^ (stock no. 013148) and AdipoQ^Cre^ (stock no. 010803) mice were purchased from Jackson Laboratories. Nrf2-deficient mice^72^ were from the same genetic background, which were kindly provided by Masayuki Yamamoto, Department of Medical Biochemistry, Tohoku University Graduate School of Medicine. Mice were males 6–8 weeks of age at the start of experiments. In this study, viWAT refers to the perigonadal visceral adipose tissue in mice. scWAT refers to inguinal subcutaneous adipose tissue in mice. For some experiments, BrdU was administered in the drinking water at 0.8 mg/ml for experiments lasting one week. High-fat diet is from Research Diets (D12492). Standard diet is from Harlan Laboratories (2018S).

### Food intake

For food intake experiments, mice were individually caged and food intake was measured by weighing the food pellets prior and after the feeding period. Kilocalories of food intake were normalized to the average body weight of the mouse at the beginning and end of the 3-day period.

### Isolation of human preadipocytes

Human SAT was obtained from healthy individuals undergoing lipoaspiration as described^41^. A total of 5 female donors were used throughout the study and the experiments were performed in accordance with the relevant guidelines and regulations by ethical committee of the Medical University of Vienna ethics committee and the General Hospital of Vienna (protocol ID EK no. 1115/2010). Informed consent was obtained from all subjects.

### Heme Oxygenase activity

Cells (approximately 3*10^6^) treated for 16 h with 20 μM hemin or vehicle alone (DMSO) were homogenized in 200 μl of a buffer containing 300 mM sucrose, 20 mM TRIS and 2 mM EDTA at a pH of 7.4 by repeated freezing/unfreezing. 90 μl of the homogenate was added to a reaction mixture containing 500 nmole NADPH in a 100 mM potassium phosphate buffer with 1 mM EDTA (assay buffer pH: 7.4), supplemented with 20 nmoles of hemin. The mixture was incubated under constant agitation at 37 °C in darkness for 30 min. The reaction was stopped by transferring the samples on ice followed by addition of 1/5 volume of saturated KCl. The formed bilirubin was extracted into benzene (4× the assay volume) by vortexing (3 × 30 s) and the organic phase was harvested by centrifugation (250 × g). Bilirubin concentration of the extracts was determined by photo spectroscopy (U-3000, Hitachi, Tokyo, Japan), scanning twice the absorption between 600 and 400 nm using the following settings: slit: 2 nm, 120 nm/min, PMT: autogain, high resolution. The difference in absorption was determined between 450 and 520 nm and the calculation of bilirubin concentration was obtained using a standard calibration curve, generated by adding known amounts of bilirubin to the assay buffer. Protein concentrations of cell homogenates were determined using the Coomassie Brilliant Blue assay (Bradford). HO activity was calculated as nmole bilirubin formed per mg protein per 30 min.

### Whole-mount immunofluorescence

Whole-mount visceral fat samples were processed and stained as described^73^. Briefly, 6–7 week old male C57BL6/J mice (three per group) were fed a SD or HFD for three days. Thereafter, fat samples were minced into 1–2 mm pieces and permeabilized for 30 min at room temperature with 0,5% PBS-T. After 3 washes with 0,05% PBS-T tissue pieces were incubated in 5% normal goat serum (Dako) in PBS to block unspecific binding sites. After blocking, tissues were incubated overnight at 4 °C with primary antibodies diluted in PBS-T (rabbit anti-HO-1; 1:500; ab13243, Abcam and rat anti-CD24; 1:500; Cat.-Nr. 101801, Biolegend). The following secondary antibodies were used for immunofluorescence staining: fluorescein anti-rat IgG (Cat.-Nr. FI-4001, Vector Laboratories), Alexa Fluor-594 goat anti-rabbit IgG (Cat.-Nr. A11037, Life Technologies). For control experiments, the primary antibody was substituted with preimmune serum. Stained tissues were incubated with Celltracker Green Bodipy Dye (Cat.-Nr. C2102, Thermo Scientific) to visualize lipid droplets for 10 minutes at room temperature before mounting in ibiTreat μ-dishes (ibidi GmbH) and subjected to confocal imaging analysis on a LSM 700 Laser Scanning Microscope (Zeiss).

### Flow cytometry analysis of cell proliferation

Isolation of adipose tissue stromal cells was performed essentially as described^20^. Briefly, adipose tissue was excised, minced, and digested in Hank’s Balanced Salt Solution (HBSS) (Sigma no. H8264) containing 3% BSA and 0.8 mg/ml Collagenase Type 2 (Worthington Biochemical; LS004174) for 75 min in a shaking water bath at 37 °C. The mixture was then filtered through a 40 μm filter, and filtered cells were pelleted and washed in HBSS containing 3% BSA. For Ki67 analysis, cells were stained with the following antibodies at room temperature: CD45 PE-Cy7 (Biolegend; clone 30-F11), CD31 PE-Cy7 (Biolegends; clone 390), CD29 Alexa Fluor 700 (BioLegend; clone HMβ1-1), CD34 Alexa Fluor 647 (BioLegend; clone MEC14.7) and CD140a PE (Biolegend; clone APA5). Anti-Ki67 FITC antibody (eBioscience, clone SOLA15) was used overnight at 4 °C. Cells were washed, fixed and permeabilized using the Fixation/Permeabilization kit (eBioscience) as per manufacturer’s protocol. All antibody incubations and washing steps were performed in HBSS with 3% BSA. Following antibody incubation, samples were analyzed on a BD LSR Fortessa analyzer. Data analysis was performed using BD FACS Diva software (BD Biosciences).

### Isolation of AP cells by bead separation

sc or viWAT was digested, filtered, and washed as described for flow cytometry analysis. Adipose precursors were then isolated as per the manufacturer’s MACS protocol (Miltenyi) using first negative selection of CD45 and CD31 followed by positive selection for SCA-1. Briefly, cell suspensions were incubated with the Non-Adipocyte Progenitor Depletion Cocktail, washed and passed over a lineage depletion column. The flow-through was collected, washed, and incubated with the Adipocyte Progenitor Isolation Cocktail. Cells were then washed and passed over a lineage selection column. Retained cells were eluted, counted, and either lysed directly into RLT lysis buffer for RNA isolation and subsequent Q-PCR, or RIPA buffer for protein isolation and subsequent western blotting, or cultured as previously described (Ohno *et al*., 2012). Briefly, APs were expanded in DMEM F12 with 10% fetal bovine serum (GIBCO) in 6 cm tissue culture plates at low confluency until ready for use. Cells were replated at 1 × 10^5^ cells per well in 24-well plates and treated as indicated. After treatment, cells were thoroughly washed, and RNA isolated using RNeasy mini kit (Qiagen) as per manufacturer’s protocol.

### AP stimulation with fatty acids

Bead isolated APs from male C57BL/6 J and NRF2 knockout mice were seeded in 24 Well plates and cultured until they reached confluency. Cells were then treated with either palmitic, oleic or linoleic acid at a concentration of 250 μM for 16 hours. Fatty acid/BSA conjugates were prepared by dissolving the fatty acid powder in 0.1 M NaOH at 70 °C to generate a 100 mM stock solution. A 10 mM solution was prepared by mixing the stock solution with a 5% (w/v) free fatty acid free BSA solution in double distilled water. Finally, the 10 mM solution was further diluted in serum free medium to obtain the final concentration of 1.0 mM fatty acid/0.5% BSA.

### Adenovirus experiments

Generation of AdHO-1 and AdLacZ was described previously^24^. In brief, Primary hASC were infected with 30 plaque forming units (pfu)/cell.

### Gene expression profiling

#### Adipose precursor cell screen

Each of the eight microarray samples represent RNA isolated from APs derived from scWAT or viWAT from SD- or HFD fed C57BL/6 J mice (at age 7–8 weeks) representing pooled samples from 3 mice. Data were imported and preprocessed using R and Bioconductor, specifically the ‘oligo’ package version ‘1.34.2’ and ‘pd.mogene.1.0.st.v1’ version ‘3.14.1’. Data were preprocessed using quantile normalization to account for latent batch effects and RMA to summarize probesets. This combination has been shown to effectively reduce batch effects and to provide good sensitivity for the detection of differentially expressed genes^74^. For each probeset we fit a linear model to the effect of CRE treatment. An empirical bayes procedure was used to exploit information across different probesets by shrinking means and standard deviations toward common values. Non-specific filtering was applied to reduce the number of statistical hypothesis tests. Only probesets with log2 expressions above 7 in at least 3 samples, and a mean absolute deviation in the upper terzile were selected. Among the remaining probesets we only selected those that had a unique Gene-Symbol annotation using Bioconductor package ‘mogene10sttranscriptcluster.db’ version 8.4.0. Statistical significance of the estimated differential effects was assessed using t-tests and corresponding p-values. Multiple testing adjustment was performed using the Benjamini-Hochberg procedure, which provides control of the false discovery rate.

### Gene Set Enrichment Analysis (GSEA)

Biological insights concerning the differentially expressed genes were explored via Gene Set Enrichment Analysis (GSEA). The analysis was performed with the GSEA software^75^ (version 2.1.0) using the c2 (version 5) gene set database. In addition, a 3T3-L1 differentiation time course^31^ was used to construct gene sets resembling preadipocyte‚ ‘proliferation’ or‚ ‘terminal differentiation’, by assembling differentially expressed genes between day-2 vs. day 0 or day 7 vs. day 2, respectively^76^. Genes were first ranked based on real value using the weighted signal-to noise metric. P-values and the false discovery rate (FDR) for the enrichment score of each gene set were calculated based on 1000 gene set permutations. Enrichment plots were generated using Cytoscape 3.3.0 and the plugin Enrichment map^77^.

### Oxygen consumption assay and bioenergetic profile

Oxygen consumption rates (OCR) of bead-purified murine APs from viWAT were performed using the XF24 Flux Analyzer (Seahorse Bioscience) as reported previously^24^. In brief, bead-purified visceral APs were seeded into XF 24-well cell culture microplates. After a 24 hour recovery period cells were washed and a final volume of 630 μl buffer-free Assay Medium (Seahorse Bioscience, supplemented with 5 mM glucose (Sigma) and 1 mM sodium pyruvate (Gibco)) was added to each well prior to the experimental protocol. Cells were then kept in a CO2-free incubator at 37 °C for 1 hour. After instrument calibration, cells were transferred to the XF24 Flux Analyzer to record cellular oxygen consumption rates. Measurements were performed with repetitive cycles of 2 min mixture, 2 min wait and 4 min OCR measurement times. Injected compounds for the mitochondrial stress test were oligomycin (2 μM working concentration) to inhibit ATP synthase, followed by FCCP (1 μM working concentration) to induce mitochondrial uncoupling and rotenone/antimycin A (2 μM working concentration each) to block the mitochondrial respiratory chain. Following the assay, the medium was carefully aspirated and cellular protein content was measured using the Pierce BCA Protein Assay Kit (Thermo Scientific) to adjust for potential differences in cell numbers. For ROS-quenching experiments, APs were incubated with 10 μM buffered N-acetyl-cysteine (NAC) for 1 hour prior to otherwise unchanged experimental procedures.

### Western blot analysis

Protein concentration for the resulting lysates was determined using the BCA (bicinchoninic acid) Protein Assay kit from Pierce, and lysates were run on 10% polyacrylamide gels. PVDF membranes (GE healthcare) were incubated with following antibodies: HO-1 (Abcam), β-actin (Sigma), pAkt2-S474 and Akt2 (all Cell Signaling). Hrp-conjugated IgG secondary antibodies were used (Cell Signaling) and blots were developed with ECL Plus Western Blotting Detection System (GE Healthcare). ImageLab software (BioRad) was used for densitometric quantification.

### Adipocyte differentiation

At 2 days post-confluency preadipocytes were treated with a differentiation cocktail containing: DMEM, 10% CS, insulin (870 nM), TZD (5 μM), Dex (1 μM), and IBMX (500 μM). On day 3 of differentiation, cells were switched to differentiation cocktail excluding IBMX and Dex for remaining duration of differentiation.

### Q-PCR

Q-PCR was performed as previously published^41^. All Q-PCR data are normalized to amounts of acidic ribosomal phosphoprotein P0 (RPLP0), unless otherwise stated. Primer sequences are summarized in Supplemental Table S6.

### Oil-red O staining

Cells were fixed in 10% formalin, thereafter stained for 20 minutes with Oil-RedO (Sigma) working solution (composed of 4 parts water and 6 parts 0.6% Oil-red O dye in isopropanol). Oil-Red O was extracted using 100% isopropanol and absorbance was measured at 490 nm using a spectrophotometer.

### Retrovirus preparation and infection

Retrovirus preparation and infection were performed as described^78^. Briefly, pMSCV, LMP empty vectors, or their derivatives containing specific cDNA or shRNAmir, along with vectors containing reverse transcriptase (gag-pol) and VSV-G-expressing plasmids, were transfected into Phoenix packaging cells with the CellPhect transfection kit (Amersham Biosciences). Viral supernatant was collected 48 hrs after transfection, filtered through 0.45 μm filters, and added to target cells for 12 hrs along with 8 g/ml Polybrene. Cells were selected with 4 g/ml puromycin or 400 g/ml hygromycin to make stable lines and were maintained in media containing appropriate antibiotics.

### Construction of transgenic cell lines

Constitutive stable HO-1 knock-down in 3T3-L1 cells was generated by transduction with the microRNA (miRNA) adapted retroviral vector LMP (OpenBiosystems, Huntsville, AL, USA). shRNAmir (microRNA-adapted short hairpin RNA) against murine HO-1 generated in pSM2 vector (Open Biosystems) was subcloned into the LMP vector with XhoI and EcoRI (Invitrogen, Carlsbad, CA) restriction enzymes. Confirmation was verified by restriction site analysis and sequencing. To produce murine stem cell virus (MSCV) particles, HEK293FT cells were transiently co-transfected with a vector containing the viral packaging proteins gag and pol, a vector containing env, and either LMP, or LMP-miHO1 (LMP containing miRNA against human HO-1). Vectors containing gag, env, and pol were kind gifts from Dr. Ewan Rosen (Beth Israel Deaconess Medical Center, Harvard Medical School, Boston, MA, USA). Lipofectamine 2000 (Invitrogen) reagent was used for transfection. 48 hours after transfection, viral supernatants were collected, centrifuged at 1500 rpm for 3 min, filtered through a 0.4 μm filter, supplemented with 8 μg/ml Polybrene (Sigma), and used to infect 3T3-L1 cells. Stable integrants were selected with puromycin (5 μg/ml) over a period of 2 weeks. HO-1 knock-down was verified by western blotting. The coding region of human HO-1 cDNA (a gift of Dr. Matthias Mayerhofer, Department of Laboratory Medicine, Medical University of Vienna, Vienna, Austria) was inserted into the retroviral vector pMSCV-puro (Clontech, Mountain View, CA, USA) at the BamHI and HindIII sites within the MCS. To produce MSCV retrovirus, HEK293FT cells were transiently co-transfected with a vector containing the viral packaging proteins gag and pol, a vector containing env and MSCV or MSCV-HO1. Lipofectamine 2000 (Invitrogen) reagent was used for transfection. 48 hours after transfection, viral supernatants were collected, centrifuged at 1500 rpm for 3 min, filtered through a 0.4 μm filter, supplemented with 8 μg/ml Polybrene (Sigma), and used to infect 3T3-L1 cells. Stable cell pools transduced with either empty plasmid or plasmid carrying HO-1 cDNA were termed MSCV and MSCV-HO1, respectively. HO-1 expression was verified by western blotting.

### BrdU cell proliferation assay

BrdU uptake in 3T3-L1 gain/loss of function cells and in human SVFs was determined as previously described^41^. Briefly, untreated, LMP, miHO-1, MSCV and MSCV HO-1 were plated into 96-well culture plates at a density of 10,000 cells per well. Differentiation was initiated two days after seeding and cells were kept at 37 °C for 10 days. Proliferation was established using the Cell Proliferation ELISA, BrdU kit (Roche) according to manufacturer’s protocol. In brief, 10 μl BrdU labeling solution was added to 100 μl differentiation medium per well. Cells were then incubated at 37 °C for 2 hours, followed by the removal of labeling medium and addition of 200 μl/well FixDenat solution. After a 30 minute incubation time at RT the solution was removed by tapping. Thereafter, 100 μl/well anti-BrdU-POD working solution was added for 90 minutes at RT. Wells were washed 3 times and then 100 μl/well substrate solution was added for 15 minutes at RT. Finally, cells were treated with 25 μl/well 1 M H2SO4 stop solution. Absorbance was measured on a multimode microplate reader (Synergy 2, BioTek Instruments) at 450 nm and a reference wavelength of 690 nm.

### Nitroblue tetrazolium (NBT) assay

Bead-purified adipose precursors of 8 to 10-week-old control (Hmox1^fl/fl^) and HO-1 knockout (Hmox1^fl/fl^Pdgfra^Cre^) mice were cultured in 48-well culture plates. After a 48 hour recovery phase cells were incubated with 0.2% NBT (Sigma) in PBS for 2 hours at 37 °C. Reduced NBT was dissolved in 50% acetic acid and absorbance was measured at 560 nm. Results were normalized to individual cell counts.

### MitoSOX Red measurement

Visceral APs were bead-purified as previously described and seeded into 24 Well plates. Cells were allowed to adhere for 24 hours and afterwards incubated with 5 μM MitoSOX Red (Molecular Probes; M36008) for 15 min at 37 °C. Cells were washed off with PBS and after two further washing steps cells were resuspended in HBSS containing 3% BSA. Superoxide production was measured as fluorescence intensities at 510/580 nm.

### Histology and Adipocyte size

Hematoxylin and eosin (H&E) staining was performed on 5 μm paraffin sections. Adipocyte size distribution was determined by semi-automated morphometry. In brief, 3 fields of view of 3 different sections per animal were quantified. Visceral and subcutaneous fat pads from 4 animals per group were analyzed. Adipocyte size was measured using Adiposoft^79^.

### Adipose tissue collagen assay

Collagen was assessed using the QuickZyme Total Collagen Assay as per manufacturer’s protocol. Briefly, after 8 weeks on high fat diet ~300 mg of visceral adipose tissue was directly incubated in 6 M HCl for 20 hours at 95 °C. Tubes were then cooled to room temperature and centrifuged at 13,000 g for 10 min. 35 μl of the supernatant was added to 75 μl of assay buffer in a microplate and incubated at RT for 20 min. Afterwards 75 μl detection reagent was added to each well and the microplate was incubated for 1 hour at 60 °C. Absorbance was measured at 570 nm and collagen content was calculated using a standard curve.

### *Ex vivo* lipolytic assessment of adipose tissue

After an 8 week long HFD feeding period, visceral adipose tissue was surgically removed and washed in PBS. Fat depots were cut into 20 mg pieces and incubated in DMEM containing 2% fatty-acid-free BSA either with 10 μM isoproterenol (stimulated) or without (basal) for 1 hour at 37 °C. Fat pieces were then transferred into fresh medium and incubated for another hour. Free fatty acid release into the medium was measured using the WAKO NEFA-HR (2) kit as per manufacturer’s protocol.

### Trichrome staining

Visceral adipose tissue of obese mice was fixed in 10% formalin for at least 24 hours at 4 °C and afterwards embedded in paraffin. Blocks were cut in 10 μm slices and incubated overnight at 56 °C. Following standard dehydration steps (20 min Xylen, 2 min 100% EtOH, 2 min 95% EtOH, 2 min 70% EtOH) slices were submerged into Postmordanding solution (200 ml saturated picuis acid; 8 g mercuris chloride) for 1 hour. After a warm tap water wash slices were incubated in Verhoff’s stain (120 ml hematoxylin; 13,5 ml ferric chloride; 66,5 ml Weigerts iodine) for 8 min and washed again. Slices were stained for 5 min with Fast yellow (2 g Acid yellow 17; 1 ml glacial HAc; 200 ml dH_2_O) and washed three times with 0,5% HAc. This was followed by 6 min in Biebrich stain (1,5 g Biebrich scarlet; 1,5 g acid fuchsin; 1,5 g Ponceau 2 R; 1 ml glacial HAc; 200 ml dH_2_O) and a wash with dH_2_O. Afterwards slices were incubated for 6 min with Phosphotungstic acid (2%) and washed again with dH_2_O. Finally, slices were stained with Light green stain (4 g Light green SF; 4 g glacial HAc; 200 ml dH_2_O) for 10 min and washed three times with 0,5% HAc. Slices were washed extensively in dH_2_O, and following 2 min in 70% EtOH and 2 min in 100% EtOH they were incubated in butylacetat for 20 min. Eukitt embedded fatpad slices were imaged using an Axioimager M2 (Zeiss).

### Statistical Analysis

Data are expressed as mean ± standard error of the mean (SEM). Statistical significance was tested by Student’s t test or 2-way ANOVA where appropriate. Correlations were tested by linear regression. All figures and statistical analysis were generated using Prism 6 (GraphPad). All reported p values are two-tailed. p < 0.05 was considered to indicate statistical significance.

## Additional Information

**Accession Code:** Data sets have been deposited in the Gene expression omnibus archive as series GSE80148. A reviewer accessible link has been created and is available at http://www.ncbi.nlm.nih.gov/geo/query/acc.cgi?token=ejgdqouubpirhsv&acc=GSE80148.

**How to cite this article**: Wagner, G. *et al*. HO-1 inhibits preadipocyte proliferation and differentiation at the onset of obesity via ROS dependent activation of Akt2. *Sci. Rep.* **7**, 40881; doi: 10.1038/srep40881 (2017).

**Publisher's note:** Springer Nature remains neutral with regard to jurisdictional claims in published maps and institutional affiliations.

## Supplementary Material

Supplemental Information
