# Supplementary material for: HO-1 inhibits preadipocyte proliferation and differentiation at the onset of obesity via ROS dependent activation of Akt2
Source: Sci Rep. 2017 Jan 19;7:40881. doi: 10.1038/srep40881 (PMC5244367; doi:10.1038/srep40881)
Supplement: Supplemental Information [file srep40881-s1.pdf]

SUPPLEMENTAL INFORMATION referring to:

HO-1 inhibits preadipocyte proliferation and differentiation at the onset of obesity via ROS dependent activation of Akt2

Gabriel Wagner[1], Josefine Lindroos-Christensen[1*], Elisa Einwallner[1], Julia Husa[1], Thea-Christin Zapf[1#], Katharina Lipp[2], Sabine Rauscher[3], Marion Gröger[3], Andreas Spittler[3], Robert Loewe[2], Florian Gruber[2,9], J. Catharina Duvigneau[4], Thomas Mohr[5], Hedwig Sutterlüty-Fall[5], Florian Klinglmüller[6], Gerhard Prager[7], Berthold Huppertz[8], Jeanho Yun[10], Oswald Wagner[1], Harald Esterbauer[1] and Martin Bilban[1,3].

INVENTORY

Supplemental information includes 3 figures with legends, followed by 6 tables with legends.

Supplemental Figure S1

(A) Body weight (left panel) and fat pad weight (right panel) after 3 days of SD or HFD ($n = 6$).

(B) Analysis of gene expression by Q-PCR of WAT fractions (n = 6-8 mice).

(C) HO-1 mRNA expression in bead-isolated APs derived from scWAT of wildtype mice stimulated with 250µM palmitic acid.

(D) HO-1 mRNA expression in bead-isolated APs stimulated with 250µM palmitic (PA), oleic (OA) or linoleic acid (LA).

(E) HO-1 mRNA expression in bead-isolated APs from scWAT derived from wildtype (WT) or Nrf2-knockoutmice (Nrf2-KO) stimulated with 250µM palmitic acid.

Results are mean ± SEM. *$p < 0.05$, **$p < 0.01$, ***$p < 0.001$, ****$p<0.0001$

Figure S1

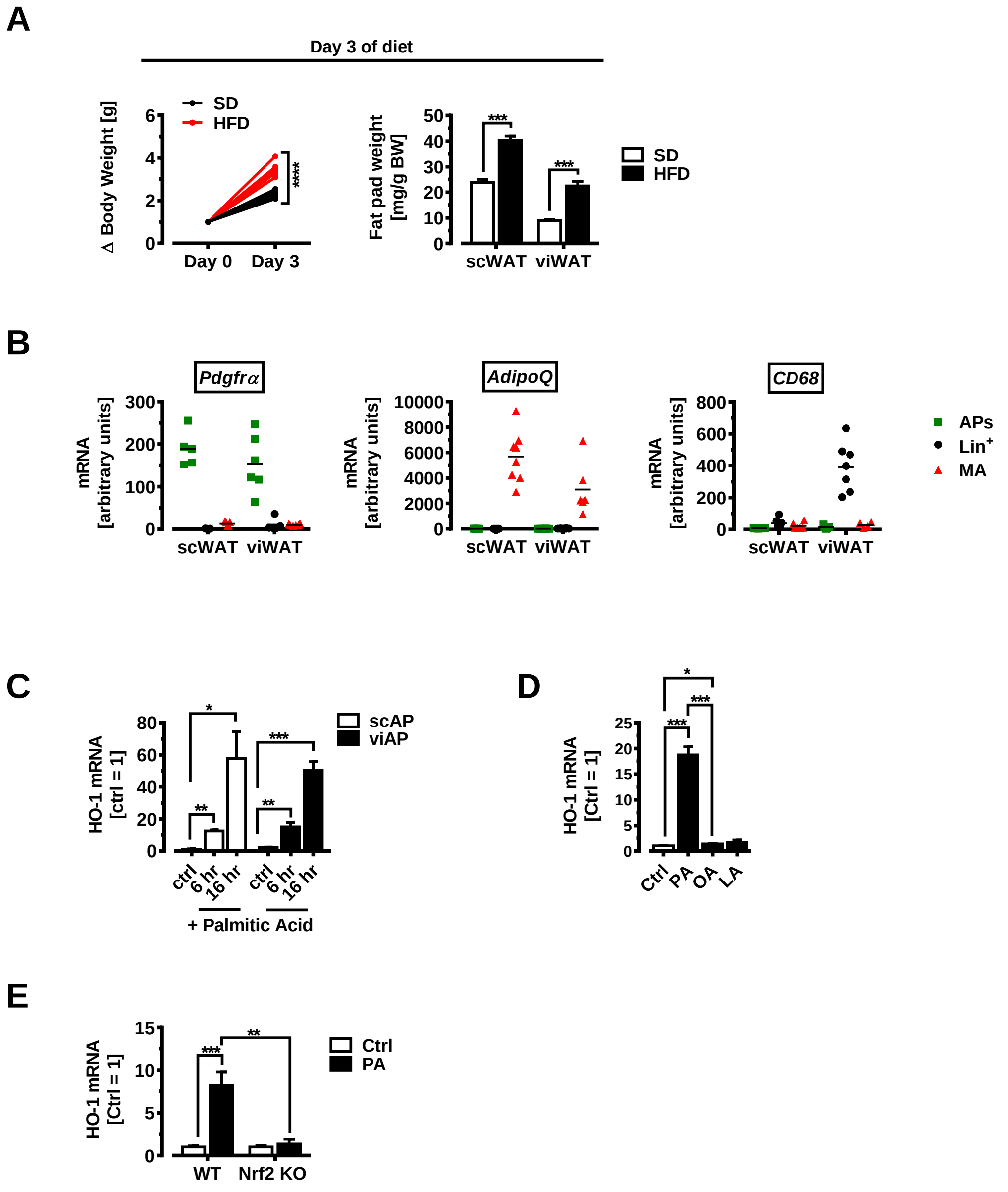
A
Day 3 of diet
Δ Body Weight [g]
SD
HFD
****
Day 0
Day 3
Fat pad weight [mg/g BW]
***
***
scWAT
viWAT
SD
HFD
B
Pdgfrα
AdipoQ
CD68
mRNA [arbitrary units]
scWAT
viWAT
APs
Lin+
MA
C
HO-1 mRNA [ctrl = 1]
scAP
viAP
ctrl
6 hr
16 hr
+ Palmitic Acid
D
HO-1 mRNA [Ctrl = 1]
Ctrl
PA
OA
LA
E
HO-1 mRNA [Ctrl = 1]
Ctrl
PA
WT
Nrf2 KO

Supplemental Figure S2

(A) Body weight of Hmox1$^{fl/fl}$ and Hmox1$^{fl/fl}$Pdgfra^{Cre} at 6 weeks of age.

(B) Gating strategy for the identification of APs in the scWAT and viWAT of mice using flow cytometry.

(C) Quantification of food intake normalized to body weight in the indicated groups of mice on SD or during the first 3 days of HFD feeding (n = 2-3).

Results are mean ± SEM. *p < 0.05, **p < 0.01, ***p < 0.001

Figure S2

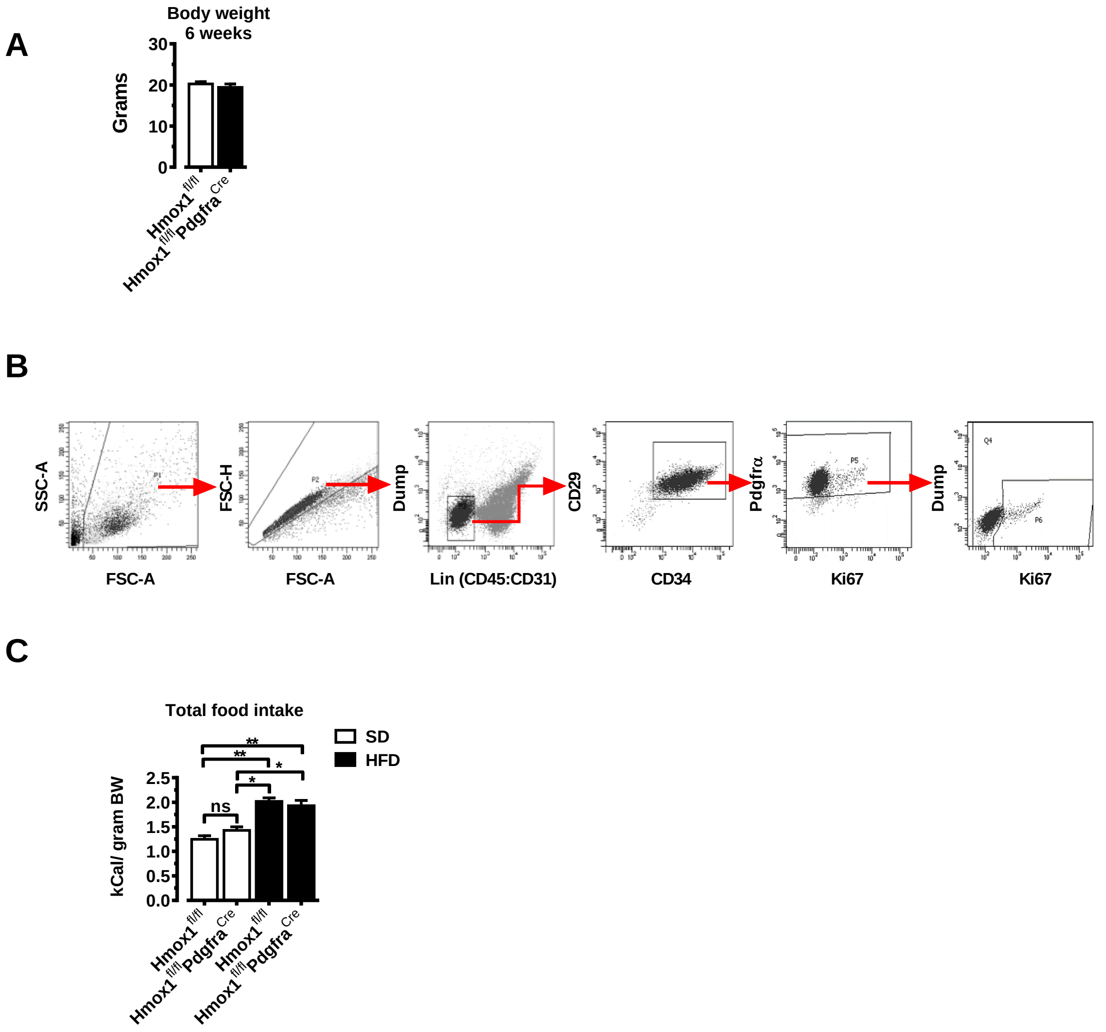

Supplemental Figure S3

(A-D) Body weight gain and weights of individual fat pads of mice fed a chow diet (A and C) or HFD (B and D) for 8 weeks (n = 10-12).

(E) Adipocyte size distribution in scWAT and viWAT of Hmox1$^{fl/fl}$ and Hmox1$^{fl/fl}$Pdgfra^{Cre} mice kept on HFD for 8 weeks, as determined by quantitative morphometry of H&E stained tissue sections (n = 6). Bar = 200µm.

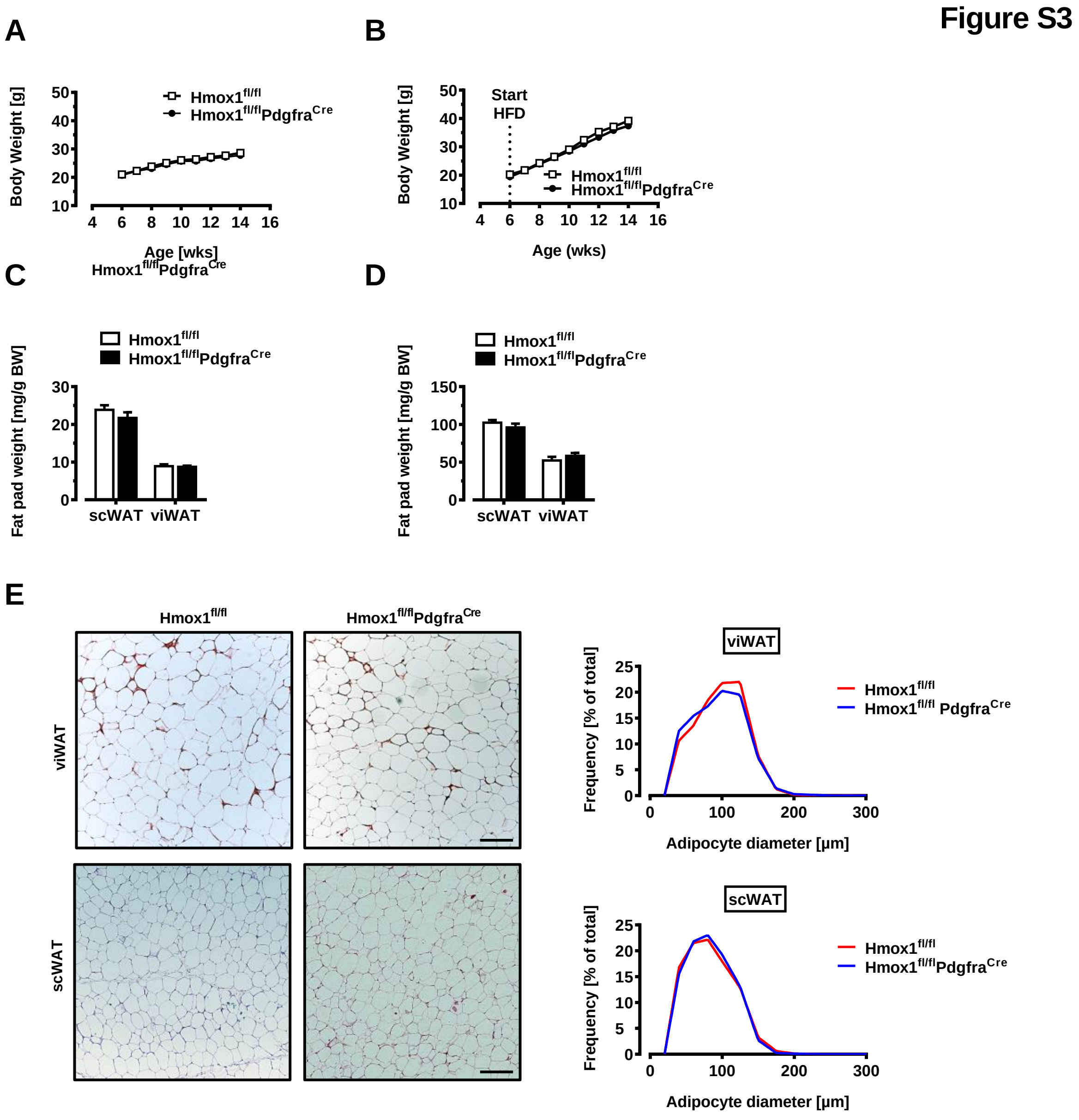

Table S1 and S2. High fat diet regulated genes in visceral and subcutaneous APs identified by DNA microarray analysis, related to Figure 1. mRNA expression profiles were generated with murine Gene Level 1.0 ST GeneChips. Mice were fed either a SD or HFD for three days and visceral and subcutaneous APs were bead purified from the stromal vascular fraction of viWAT and scWAT and lysed directly in RLT lysis buffer. Genes exhibit more than a ±2-fold change relative to controls. Genes included in the 'common signature' are highlighted in grey.

Table S3 and S4. GO analysis using DAVID of viAP and scAP gene signatures, related to Figure 1B. Mice were kept on SD or HFD for three days and bead purified APs were directly lysed before preparation of mRNA expression profiles. All P-values are derived from Fisher's exact test and pass Benjamini-Hochberg corrected $P<0.05$.

Table S1. Genes differentially expressed more than 2.0 fold between HFD and SD in viAPs.

Probe Set ID	Gene Symbol	Raw p-value	Benjamini-Hochberg corr. P-Value	Mean Fold Change HFD/SD (Log2)	SD_VI_AP1	SD_VI_AP2	HFD_VI_AP1	HFD_VI_AP2
10440091	Col8a1	5.24E-06	0.02404	2.547	6.600	6.728	9.257	9.165
10586168	Snord16a	2.94E-04	0.02404	2.362	7.629	6.895	9.633	9.615
10358565	Hmcn1	4.96E-03	0.03838	2.338	5.492	4.087	7.349	6.907
10604743	Snord61	1.54E-03	0.02748	2.321	7.011	5.954	8.943	8.665
10568714	Mki67	6.40E-05	0.02404	2.310	6.129	6.150	8.679	8.219
10547227	Ret	3.61E-05	0.02404	2.281	6.858	6.492	8.934	8.977
10554445	Prc1	2.16E-05	0.02404	2.246	5.585	5.644	8.000	7.721
10607950	G530011O06Rik	1.50E-04	0.02404	2.211	7.000	6.977	8.919	9.480
10572897	Hmox1	1.27E-04	0.02404	2.193	9.290	9.753	11.588	11.842
10390707	Top2a	2.76E-05	0.02404	2.167	5.931	5.728	8.103	7.889
10427461	Ptger4	1.80E-03	0.02798	2.161	7.267	6.267	9.087	8.768
10545588	Hk2	9.18E-06	0.02404	2.158	8.039	8.098	10.182	10.271
10497520	Ect2	2.07E-05	0.02404	2.154	6.129	6.150	8.418	8.170
10591781	Anln	3.66E-05	0.02404	2.136	6.129	6.109	8.418	8.093
10462796	Kif11	2.97E-05	0.02404	2.133	4.954	5.087	7.285	7.022
10459772	Lipg	8.96E-05	0.02404	1.981	5.524	5.129	7.349	7.267
10392484	Abca8b	8.12E-03	0.04624	1.962	8.243	6.870	9.624	9.414
10362201	Ctgf	2.19E-04	0.02404	1.940	7.925	7.508	9.814	9.498
10368289	Enpp1	1.58E-03	0.02769	1.939	6.000	5.129	7.615	7.392
10359890	Nuf2	1.14E-04	0.02404	1.935	5.000	4.644	6.870	6.644

10527920	Cyp51	2.99E-05	0.02404	1.934	6.229	6.229	8.276	8.050
10562637	Ccnb1	2.62E-04	0.02404	1.932	5.585	6.022	7.901	7.570
10398392	Mir329	7.12E-04	0.02437	1.852	6.476	7.000	8.814	8.366
10358533	Hmcn1	6.78E-03	0.04265	1.847	5.615	4.585	7.295	6.600
10594774	Ccnb2	1.97E-05	0.02404	1.846	6.539	6.508	8.422	8.317
10594251	Kif23	6.85E-05	0.02404	1.843	5.285	5.044	7.109	6.907
10432511	Racgap1	2.56E-05	0.02404	1.840	5.285	5.392	7.238	7.119
10398420	Mir382	3.19E-04	0.02404	1.826	4.907	5.087	7.077	6.570
10450374	D17H6S56E-5	8.61E-05	0.02404	1.810	6.340	6.229	8.253	7.937
10557156	Plk1	4.37E-05	0.02404	1.799	5.700	5.700	7.615	7.384
10561212	Ltbp4	4.00E-03	0.03535	1.793	10.298	9.253	11.593	11.544
10357833	Atp2b4	4.57E-03	0.03709	1.774	8.600	7.539	9.919	9.768
10454077	Taf4b	2.24E-04	0.02404	1.758	6.600	7.044	8.531	8.629
10523670	Aff1	2.63E-05	0.02404	1.754	6.340	6.229	8.061	8.017
10489891	B4galt5	7.94E-05	0.02404	1.741	7.524	7.229	9.134	9.101
10424119	Nov	8.85E-03	0.04823	1.730	8.679	7.443	9.731	9.850
10578916	Msmo1	1.29E-03	0.02653	1.697	7.077	6.426	8.611	8.285
10504178	4933409K07Rik	3.24E-03	0.03275	1.693	6.794	7.687	9.063	8.804
10354732	Hspd1	2.83E-04	0.02404	1.692	6.129	6.539	8.134	7.919
10411739	Ccnb1	4.73E-04	0.02404	1.690	5.129	5.358	7.180	6.687
10385248	Hmmr	3.26E-04	0.02404	1.681	4.807	4.807	6.728	6.248
10504148	Gm3893	2.75E-03	0.03101	1.676	6.820	7.665	9.039	8.798
10515836	Ccnb1	3.80E-04	0.02404	1.667	4.954	5.129	6.943	6.476
10497831	Ccna2	7.78E-04	0.02437	1.663	6.340	5.954	8.050	7.570
10555174	Lrrc32	2.14E-04	0.02404	1.661	8.562	8.165	9.979	10.070
10444778	Gm25128	1.87E-03	0.02830	1.655	7.170	6.392	8.401	8.472
10406676	Lhfpl2	2.17E-03	0.02937	1.654	7.644	6.870	9.033	8.788
10546760	Ddx3x	1.18E-04	0.02404	1.641	8.170	7.870	9.707	9.615
10351551	Adamts4	4.77E-03	0.03764	1.630	9.585	8.600	10.752	10.693
10363706	Jmjd1c	1.35E-04	0.02404	1.629	8.714	8.388	10.195	10.166

10490826	Zbtb10	8.63E-04	0.02440	1.628	6.285	6.883	8.290	8.134
10361807	Hivep2	3.42E-04	0.02404	1.627	7.845	7.409	9.333	9.175
10517336	Clic4	1.09E-04	0.02404	1.620	8.898	9.187	10.687	10.639
10398426	Mir485	1.04E-04	0.02404	1.612	5.833	6.022	7.644	7.435
10571530	Fat1	7.58E-04	0.02437	1.608	7.615	7.170	9.190	8.811
10482762	Idi1	2.21E-03	0.02957	1.603	6.392	5.644	7.741	7.500
10487252	Gabpb1	1.37E-04	0.02404	1.598	6.109	6.340	7.931	7.714
10398400	Mir543	1.02E-04	0.02404	1.589	5.129	5.285	6.907	6.687
10354432	Myo1b	4.74E-03	0.03749	1.586	6.524	5.672	7.901	7.468
10497503	Kpna2	6.01E-04	0.02411	1.584	6.476	6.000	7.943	7.700
10504123	4933409K07Rik	4.40E-03	0.03643	1.581	6.845	7.748	8.994	8.762
10504125	4933409K07Rik	4.40E-03	0.03643	1.581	6.845	7.748	8.994	8.762
10398390	Mir323	1.50E-03	0.02748	1.573	5.426	5.977	7.484	7.066
10465895	Fads2	5.41E-03	0.03961	1.573	8.504	7.524	9.598	9.576
10594161	Arih1	7.29E-05	0.02404	1.568	8.791	8.589	10.282	10.234
10357579	Mapkapk2	8.08E-05	0.02404	1.549	7.119	6.907	8.581	8.543
10504169	4933409K07Rik	1.52E-03	0.02748	1.548	6.954	7.592	8.940	8.704
10441987	Chd1	8.52E-05	0.02404	1.547	7.229	7.451	8.880	8.895
10467420	Pdlim1	7.70E-04	0.02437	1.545	7.741	7.190	9.047	8.974
10392284	Kpna2	1.90E-04	0.02404	1.545	6.285	6.087	7.870	7.592
10492195	Tsc22d2	5.63E-05	0.02404	1.540	8.451	8.313	9.947	9.896
10435271	Heg1	3.02E-03	0.03197	1.531	10.534	9.723	11.664	11.656
10400095	Ifrd1	5.88E-05	0.02404	1.531	10.006	10.011	11.615	11.464
10565962	P2ry2	4.08E-04	0.02404	1.530	7.794	7.349	9.082	9.122
10563112	Snord33	2.49E-04	0.02404	1.518	8.443	8.077	9.758	9.798
10428536	Trps1	7.08E-03	0.04346	1.515	5.833	4.954	7.160	6.658
10538832	Mad2l1	7.49E-04	0.02437	1.505	5.931	5.492	7.366	7.066
10579969	Zfp330	1.61E-03	0.02769	1.503	5.585	6.209	7.516	7.285
10583732	Ldlr	3.69E-03	0.03452	1.502	8.353	7.516	9.449	9.424
10581266	Tppp3	1.18E-03	0.02592	1.490	9.695	9.095	10.911	10.860

10371770	Gas2l3	3.55E-04	0.02404	1.489	4.954	5.322	6.714	6.539
10504121	4933409K07Rik	4.09E-03	0.03564	1.489	6.907	7.728	8.922	8.690
10384150	Purb	3.22E-03	0.03271	1.486	6.833	6.066	8.044	7.827
10532711	Cmklr1	7.35E-03	0.04420	1.484	8.555	7.585	9.672	9.435
10389526	Cltc	7.37E-05	0.02404	1.482	9.326	9.170	10.755	10.706
10493640	Nup210l	2.69E-04	0.02404	1.478	5.322	5.585	7.055	6.807
10546706	Rybp	2.94E-03	0.03169	1.476	6.714	7.476	8.629	8.512
10512327	4933409K07Rik	1.36E-03	0.02693	1.475	6.977	7.562	8.845	8.644
10456745	Smad7	4.76E-05	0.02404	1.475	6.524	6.555	8.000	8.028
10596394	Col6a6	3.52E-03	0.03389	1.473	8.690	7.883	9.738	9.780
10411235	Iqgap2	1.59E-03	0.02769	1.464	7.820	7.180	8.945	8.983
10523647	Aff1	1.08E-04	0.02404	1.463	8.050	8.209	9.667	9.518
10388782	Snord42a	1.94E-03	0.02830	1.460	7.852	7.200	9.077	8.895
10368409	Lama2	1.25E-04	0.02404	1.454	10.006	10.213	11.619	11.507
10404686	Bmp6	4.50E-03	0.03676	1.453	7.577	6.728	8.633	8.577
10507594	Slc2a1	7.95E-03	0.04581	1.449	8.543	7.555	9.447	9.549
10571788	Vegfc	3.60E-03	0.03419	1.448	7.484	6.781	8.768	8.392
10355582	Usp37	1.58E-04	0.02404	1.445	6.170	5.907	7.508	7.459
10421877	Diap3	2.97E-04	0.02404	1.442	5.585	5.907	7.267	7.109
10556509	Spon1	6.36E-03	0.04167	1.440	8.468	7.547	9.392	9.502
10413059	Vcl	6.01E-03	0.04108	1.437	8.262	7.392	9.399	9.129
10477187	Tpx2	4.80E-03	0.03767	1.431	6.066	5.459	7.492	6.895
10577508	Ckap2	1.02E-04	0.02404	1.422	5.492	5.555	7.033	6.858
10364856	Dot1l	8.13E-05	0.02404	1.419	7.229	7.087	8.589	8.566
10607283	Maged2	1.49E-03	0.02748	1.415	8.170	7.577	9.342	9.236
10416215	Loxl2	1.93E-03	0.02830	1.411	8.861	9.508	10.613	10.578
10454709	Kif20a	2.73E-04	0.02404	1.411	6.190	5.954	7.600	7.366
10526853	Fam20c	4.03E-03	0.03546	1.399	7.707	6.919	8.687	8.738
10412038	Zswim6	1.15E-04	0.02404	1.398	7.313	7.209	8.577	8.741
10375941	Vdac1	6.12E-04	0.02411	1.393	8.234	7.827	9.512	9.335

10384656	B3gnt2	1.85E-03	0.02822	1.390	6.629	6.066	7.877	7.600
10389606	Prr11	2.49E-04	0.02404	1.387	5.644	5.358	6.943	6.833
10491721	Spry1	2.32E-04	0.02404	1.379	7.531	7.435	9.000	8.725
10590031	Itga9	6.12E-03	0.04136	1.377	8.039	7.170	9.014	8.948
10428604	Tnfrsf11b	9.45E-03	0.04964	1.374	7.285	6.358	8.358	8.033
10369932	Susd2	3.79E-03	0.03485	1.373	9.375	8.622	10.322	10.422
10428157	Rnf19a	2.82E-04	0.02404	1.373	7.500	7.219	8.807	8.658
10489204	Tgm2	8.53E-03	0.04719	1.372	8.655	7.728	9.676	9.451
10504139	Gm3893	3.35E-03	0.03318	1.369	6.966	7.693	8.728	8.669
10504957	Smc2	2.39E-03	0.02976	1.367	6.087	5.426	7.129	7.119
10528268	Ptpn12	7.31E-05	0.02404	1.365	8.371	8.418	9.785	9.735
10453512	Kpna2	4.68E-04	0.02404	1.360	6.476	6.129	7.748	7.577
10404049	Hist1h3a	6.36E-03	0.04167	1.360	9.025	8.165	10.015	9.895
10358625	Hmcn1	2.08E-03	0.02888	1.360	5.807	5.248	7.033	6.741
10515427	Gm26330	9.39E-05	0.02404	1.359	4.907	5.000	6.358	6.267
10382243	Gna13	2.65E-03	0.03052	1.356	8.129	8.804	9.828	9.817
10555695	Rrm1	4.17E-04	0.02404	1.351	7.516	7.170	8.752	8.637
10369792	Arid5b	1.33E-03	0.02692	1.350	8.384	7.839	9.482	9.441
10474243	Cstf3	3.50E-03	0.03389	1.345	6.000	5.285	7.044	6.931
10475350	Serf2	2.48E-03	0.03003	1.345	6.755	7.322	8.547	8.219
10569707	Myadm	4.23E-03	0.03599	1.342	10.159	9.418	11.220	11.041
10494405	Hist2h3c2	4.43E-03	0.03651	1.341	9.170	8.405	10.180	10.078
10398360	Gm23508	1.57E-03	0.02769	1.341	6.409	6.304	7.977	7.418
10467003	Ppp1r2	1.05E-04	0.02404	1.340	8.669	8.618	10.048	9.919
10376778	Mfap4	4.67E-04	0.02404	1.337	8.539	8.229	9.825	9.617
10542079	Foxm1	1.06E-03	0.02523	1.337	5.615	5.555	7.170	6.672
10475990	Slc20a1	7.75E-05	0.02404	1.331	6.524	6.492	7.827	7.852
10584628	Thy1	9.03E-03	0.04848	1.329	8.520	9.447	10.254	10.372
10413047	Plau	2.86E-04	0.02404	1.324	9.844	9.591	11.117	10.966
10606436	Hmgn5	5.56E-04	0.02411	1.324	5.248	5.248	6.768	6.375

10426689	Spats2	5.00E-04	0.02404	1.323	7.209	6.833	8.353	8.335
10501608	Vcam1	1.35E-04	0.02404	1.322	8.785	8.768	10.186	10.011
10513208	Svep1	1.19E-03	0.02592	1.322	10.374	9.920	11.586	11.351
10406254	Ell2	6.16E-04	0.02411	1.318	6.209	6.570	7.801	7.615
10487945	Gpcpd1	9.16E-04	0.02446	1.315	6.285	5.907	7.547	7.276
10504172	4933409K07Rik	3.78E-03	0.03485	1.315	6.989	7.707	8.687	8.640
10548563	Ptp4a1	4.42E-04	0.02404	1.315	10.312	9.964	11.487	11.418
10526133	Rabgef1	2.23E-04	0.02404	1.313	7.637	7.401	8.877	8.788
10355984	Serpine2	5.37E-03	0.03953	1.311	9.514	8.725	10.471	10.390
10451710	Rftn1	9.39E-03	0.04942	1.310	7.827	6.943	8.839	8.551
10395163	Lamb1	1.97E-03	0.02847	1.310	10.384	9.794	11.419	11.378
10595000	Tmod3	8.53E-04	0.02437	1.306	8.248	7.807	9.377	9.290
10454546	Map3k2	1.60E-04	0.02404	1.306	6.966	6.833	8.276	8.134
10403413	Idi1	2.56E-03	0.03008	1.303	6.150	5.555	7.267	7.044
10529425	Nop14	1.42E-03	0.02722	1.301	7.679	7.160	8.672	8.768
10428310	Azin1	5.43E-04	0.02411	1.300	8.214	7.864	9.409	9.269
10491300	Skil	2.29E-04	0.02404	1.299	7.622	7.375	8.811	8.785
10419323	Dlgap5	9.23E-04	0.02446	1.299	5.285	5.459	6.883	6.459
10568529	Ikzf5	1.97E-03	0.02846	1.295	6.870	6.322	7.989	7.794
10400057	Arl4a	6.24E-03	0.04150	1.289	7.539	6.728	8.418	8.426
10412466	Hmgcs1	7.19E-04	0.02437	1.287	8.200	7.788	9.292	9.269
10492558	Smc4	1.09E-03	0.02531	1.286	6.658	7.011	8.281	7.960
10410477	Adamts16	2.77E-04	0.02404	1.285	6.833	6.781	7.960	8.224
10344897	Sulf1	6.65E-04	0.02411	1.284	8.778	8.418	9.967	9.796
10457872	Slc39a6	4.40E-04	0.02404	1.283	6.954	7.276	8.349	8.447
10463068	Ptp4a1	4.57E-04	0.02404	1.281	10.333	10.011	11.511	11.397
10534667	Serpine1	2.44E-04	0.02404	1.280	10.576	10.404	11.859	11.682
10365286	Eid3	2.99E-04	0.02404	1.280	5.781	5.907	7.248	7.000
10523281	Sept11	1.34E-03	0.02692	1.275	8.830	8.362	9.964	9.776
10429140	Ndrg1	1.16E-04	0.02404	1.274	8.922	8.997	10.195	10.273

10389373	Appbp2	6.88E-03	0.04294	1.273	8.600	7.807	9.587	9.366
10358717	1700025G04Rik	9.98E-05	0.02404	1.272	8.604	8.626	9.914	9.860
10506736	Magoh	1.85E-04	0.02404	1.269	7.366	7.435	8.762	8.577
10374485	Peli1	7.40E-04	0.02437	1.266	7.340	7.741	8.778	8.836
10565018	Iqgap1	1.69E-04	0.02404	1.263	8.585	8.443	9.830	9.725
10560709	Pvr	1.61E-04	0.02404	1.263	8.379	8.384	9.560	9.728
10438911	Atp13a3	1.66E-04	0.02404	1.262	7.000	7.160	8.375	8.308
10547469	Wnk1	2.11E-03	0.02890	1.260	6.109	6.600	7.762	7.468
10605256	Flna	9.42E-03	0.04953	1.260	9.234	8.375	10.181	9.947
10576661	Itgb1	2.69E-04	0.02404	1.257	9.964	9.780	11.212	11.045
10487040	Fbn1	1.74E-03	0.02774	1.254	11.725	11.192	12.740	12.685
10600836	Msn	5.45E-04	0.02411	1.251	10.050	9.702	11.152	11.102
10463211	Pi4k2a	3.75E-04	0.02404	1.251	7.044	7.340	8.435	8.451
10376885	Snord49b	1.07E-04	0.02404	1.246	8.366	8.371	9.592	9.637
10408202	Hist1h3e	5.49E-03	0.03981	1.246	8.969	8.238	9.937	9.762
10366409	Zfc3h1	7.61E-04	0.02437	1.244	6.600	6.768	8.109	7.748
10446928	Ltbp1	2.23E-04	0.02404	1.242	8.611	8.435	9.822	9.707
10508986	Stmn1	8.19E-04	0.02437	1.241	6.392	6.066	7.592	7.349
10571214	Rnf122	1.75E-04	0.02404	1.239	8.392	8.243	9.520	9.594
10424349	Sqle	4.60E-03	0.03712	1.239	6.895	6.190	7.748	7.814
10594315	Fem1b	6.41E-04	0.02411	1.234	8.022	7.658	9.093	9.055
10594988	Mapk6	1.32E-04	0.02404	1.229	9.209	9.292	10.454	10.505
10463355	Scd2	2.14E-04	0.02404	1.228	9.611	9.506	10.869	10.705
10458663	Dpysl3	1.02E-03	0.02494	1.224	8.637	8.257	9.775	9.568
10441902	Smoc2	2.66E-04	0.02404	1.224	9.535	9.320	10.687	10.615
10565547	Pcf11	3.25E-04	0.02404	1.222	8.028	8.238	9.430	9.281
10572747	Tpm4	2.97E-04	0.02404	1.221	9.752	9.589	10.982	10.801
10587266	Gclc	6.14E-04	0.02411	1.221	7.833	7.508	8.960	8.823
10416657	Elf1	1.41E-04	0.02404	1.221	7.190	7.295	8.451	8.476
10360589	Ahctf1	1.35E-04	0.02404	1.218	7.349	7.304	8.585	8.504

10469575	Ptp4a1	2.69E-04	0.02404	1.216	10.107	9.898	11.260	11.177
10463410	Fam178a	4.02E-04	0.02404	1.216	5.728	5.524	6.943	6.741
10586744	Anxa2	2.15E-04	0.02404	1.215	9.872	10.051	11.205	11.149
10540072	Ppp1r2	2.83E-04	0.02404	1.215	8.392	8.205	9.581	9.445
10586246	Dennd4a	2.32E-03	0.02964	1.213	6.109	6.555	7.714	7.375
10595604	Syncrip	2.30E-04	0.02404	1.212	7.907	7.983	9.248	9.066
10353707	Ptp4a1	2.41E-04	0.02404	1.212	10.181	9.984	11.322	11.267
10363173	Gja1	2.15E-04	0.02404	1.209	9.441	9.326	10.664	10.522
10489878	Ptgis	8.31E-03	0.04663	1.209	9.202	8.388	9.966	10.042
10521972	Pcdh7	1.08E-03	0.02523	1.207	7.665	7.443	8.945	8.577
10501265	Gnai3	3.89E-04	0.02404	1.207	8.224	7.966	9.351	9.253
10389451	Med13	3.70E-04	0.02404	1.205	8.322	8.520	9.716	9.535
10392087	Ccdc47	1.16E-03	0.02581	1.204	7.022	6.600	7.954	8.077
10375980	Aff4	2.12E-04	0.02404	1.203	9.192	9.020	10.326	10.291
10485405	Cd44	5.45E-04	0.02411	1.199	7.484	7.267	8.693	8.455
10518361	Smarca5	3.03E-04	0.02404	1.199	7.735	7.531	8.889	8.775
10510700	Gpr153	1.16E-03	0.02581	1.197	8.170	8.134	9.566	9.132
10359917	Hsd17b7	2.46E-04	0.02404	1.196	7.055	6.895	8.229	8.114
10467489	Ptp4a1	2.85E-04	0.02404	1.196	10.354	10.142	11.477	11.412
10401244	Actn1	1.73E-03	0.02774	1.196	7.077	7.508	8.615	8.362
10541496	Mfap5	1.24E-03	0.02607	1.196	11.773	11.330	12.770	12.724
10411332	Hmgcr	6.56E-04	0.02411	1.194	7.451	7.109	8.447	8.500
10567043	Rras2	5.88E-03	0.04093	1.192	7.562	6.858	8.492	8.313
10356194	Trip12	2.47E-04	0.02404	1.192	8.504	8.349	9.678	9.558
10497090	Ankrd13c	1.25E-03	0.02616	1.191	7.401	6.966	8.418	8.331
10396170	Frmd6	2.53E-03	0.03008	1.190	9.020	8.459	9.935	9.923
10422227	Spry2	2.51E-03	0.03008	1.184	7.858	7.304	8.785	8.745
10542355	Emp1	2.91E-04	0.02404	1.183	11.477	11.651	12.812	12.682
10526961	Mafk	1.12E-03	0.02540	1.182	7.913	8.317	9.238	9.355
10399360	Rhob	1.85E-03	0.02822	1.181	9.234	8.765	10.269	10.091

10347748	Utp14b	4.25E-04	0.02404	1.180	5.907	6.170	7.257	7.180
10467258	Myof	7.15E-03	0.04361	1.180	8.852	8.103	9.725	9.591
10380773	Arhgap23	6.77E-04	0.02411	1.180	7.129	6.794	8.180	8.103
10436372	Dcbld2	3.34E-03	0.03310	1.177	8.243	7.651	9.180	9.069
10542335	Gprc5a	8.69E-04	0.02440	1.175	7.600	7.322	8.508	8.765
10447056	Qpct	9.40E-04	0.02446	1.175	9.011	9.377	10.438	10.301
10496771	Mcoln2	3.29E-03	0.03296	1.175	5.807	6.358	7.375	7.140
10398362	AF357355	2.23E-03	0.02957	1.173	7.468	7.858	9.014	8.658
10362538	Lama4	4.65E-04	0.02404	1.173	10.254	9.972	11.296	11.274
10354389	Slc39a10	1.55E-04	0.02404	1.171	7.000	7.077	8.209	8.209
10586254	Dennd4a	8.36E-04	0.02437	1.170	6.728	6.687	8.061	7.693
10561461	Samd4b	3.50E-04	0.02404	1.169	8.308	8.071	9.353	9.364
10354897	Trak2	3.05E-04	0.02404	1.165	7.768	7.644	8.957	8.785
10505092	Rad23b	1.42E-04	0.02404	1.165	8.129	8.145	9.301	9.301
10345442	Hs6st1	4.09E-04	0.02404	1.163	7.276	7.044	8.267	8.379
10346634	Nop58	3.92E-04	0.02404	1.163	9.464	9.219	10.533	10.475
10500813	Hipk1	8.44E-03	0.04696	1.162	9.147	8.384	10.018	9.838
10412421	Zfp131	2.38E-04	0.02404	1.160	6.807	6.741	8.011	7.858
10522712	Rest	1.87E-04	0.02404	1.158	6.476	6.443	7.562	7.672
10447190	Plekhh2	6.99E-03	0.04327	1.157	7.972	7.276	8.904	8.658
10400191	Strn3	4.60E-04	0.02404	1.157	7.607	7.468	8.811	8.577
10423109	Adamts12	8.58E-04	0.02437	1.156	9.807	10.157	11.190	11.086
10377319	Myh10	4.36E-04	0.02404	1.154	6.977	6.977	8.262	8.000
10425283	Maff	4.71E-04	0.02404	1.154	7.392	7.119	8.418	8.401
10502419	Rap1gds1	3.45E-04	0.02404	1.151	6.895	6.687	7.983	7.901
10518147	Pdpn	4.12E-03	0.03570	1.151	10.174	9.553	10.984	11.044
10358816	Lamc1	5.78E-04	0.02411	1.149	11.083	10.812	12.163	12.031
10345930	Tpp2	3.26E-04	0.02404	1.149	6.728	6.555	7.852	7.728
10511865	Ptges3	2.40E-04	0.02404	1.147	9.087	8.994	10.253	10.124
10396278	Daam1	1.89E-03	0.02830	1.145	6.820	6.426	7.907	7.629

10386455	Rasd1	3.28E-03	0.03296	1.145	7.066	6.508	7.858	8.006
10441509	Ppp1r2	2.18E-04	0.02404	1.144	8.555	8.527	9.752	9.618
10440534	Adamts5	2.12E-04	0.02404	1.144	10.670	10.764	11.905	11.816
10469457	Plxdc2	1.01E-03	0.02494	1.143	10.883	10.500	11.834	11.834
10354472	Gls	6.85E-04	0.02411	1.142	8.455	8.778	9.768	9.750
10375382	Clint1	1.90E-04	0.02404	1.142	8.883	8.916	10.090	9.993
10456490	Cep192	1.50E-03	0.02748	1.141	5.755	5.358	6.794	6.600
10440388	Hspa13	3.67E-04	0.02404	1.139	7.358	7.443	8.644	8.435
10385790	Hspa4	2.40E-03	0.02976	1.138	8.439	7.943	9.403	9.255
10502299	Nfkb1	4.13E-04	0.02404	1.138	8.577	8.409	9.719	9.543
10406626	Homer1	1.67E-03	0.02774	1.137	6.340	5.931	7.375	7.170
10535956	Stard13	5.14E-03	0.03887	1.137	6.658	6.109	7.700	7.340
10531181	Adamts3	3.36E-04	0.02404	1.136	5.931	5.781	7.066	6.919
10476740	Slc24a3	1.26E-03	0.02626	1.135	7.426	7.033	8.304	8.426
10599781	Htatsf1	2.06E-03	0.02887	1.133	7.160	6.687	8.119	7.994
10494662	Ywhah	1.61E-03	0.02772	1.132	8.919	8.500	9.922	9.762
10351825	Tagln2	5.28E-04	0.02411	1.132	9.480	9.202	10.480	10.467
10516064	Mfsd2a	2.00E-04	0.02404	1.132	5.524	5.585	6.644	6.728
10565873	Ppme1	9.20E-04	0.02446	1.130	7.349	6.989	8.290	8.308
10513818	Stmn1	6.05E-04	0.02411	1.130	6.340	6.190	7.524	7.267
10515072	Rnf11	6.03E-04	0.02411	1.126	6.966	7.190	8.109	8.299
10439005	Ppp1r2	2.43E-04	0.02404	1.124	7.392	7.313	8.535	8.418
10602020	Tbc1d8b	3.01E-03	0.03192	1.124	6.392	6.895	7.877	7.658
10601360	Atp7a	7.75E-03	0.04539	1.123	7.119	6.426	8.017	7.775
10495763	Gclm	4.93E-04	0.02404	1.121	6.954	6.700	7.977	7.919
10452648	Emilin2	2.72E-04	0.02404	1.120	9.414	9.262	10.431	10.484
10375002	Cpeb4	3.30E-03	0.03296	1.118	7.209	7.665	8.718	8.392
10500685	Atp1a1	2.00E-04	0.02404	1.117	8.768	8.683	9.850	9.834
10384579	Ugp2	5.12E-03	0.03882	1.116	8.738	8.098	9.555	9.514
10521950	Stim2	3.30E-04	0.02404	1.113	7.600	7.781	8.775	8.833

10459071	Smim3	1.68E-03	0.02774	1.113	5.248	5.644	6.459	6.658
10494402	Hist2h3c2	4.26E-03	0.03604	1.110	8.543	7.954	9.420	9.297
10474181	Abtb2	2.37E-04	0.02404	1.110	6.570	6.476	7.592	7.672
10354704	Sf3b1	2.37E-04	0.02404	1.109	9.375	9.377	10.547	10.424
10556005	Ilk	2.64E-03	0.03052	1.109	9.377	8.898	10.341	10.152
10508115	Stk40	1.40E-03	0.02721	1.109	8.331	7.925	9.269	9.205
10544837	Tril	4.20E-03	0.03591	1.109	7.629	7.044	8.508	8.384
10468992	Frmd4a	5.10E-04	0.02404	1.109	6.820	6.943	8.103	7.877
10360972	Kcnk2	2.54E-03	0.03008	1.108	6.340	5.833	7.180	7.209
10462752	Btaf1	1.89E-03	0.02830	1.108	7.140	6.755	8.180	7.931
10346722	Nbeal1	3.84E-04	0.02404	1.105	6.476	6.476	7.687	7.476
10598723	Ddx3x	4.00E-04	0.02404	1.105	10.033	10.250	11.250	11.243
10498599	Ift80	2.25E-03	0.02957	1.105	6.044	5.672	7.119	6.807
10491599	4932438A13Rik	4.51E-03	0.03676	1.105	5.700	5.285	6.820	6.375
10357280	Insig2	2.43E-03	0.02985	1.104	7.109	6.615	7.994	7.937
10567229	Smg1	1.57E-03	0.02769	1.103	7.000	6.615	8.000	7.820
10354494	Nab1	9.36E-04	0.02446	1.103	8.098	7.794	9.132	8.966
10594636	Ppp1r2	2.65E-04	0.02404	1.102	8.397	8.388	9.564	9.424
10430302	Csf2rb2	8.74E-03	0.04781	1.099	6.977	6.304	7.895	7.585
10398396	Mir679	1.87E-04	0.02404	1.098	7.000	7.000	8.098	8.098
10493555	Kcnn3	6.82E-03	0.04282	1.097	7.119	6.524	8.087	7.748
10511069	Gnb1	3.15E-04	0.02404	1.094	10.213	10.103	11.316	11.189
10414093	Glud1	2.04E-03	0.02887	1.093	8.801	9.262	10.128	10.122
10357630	Srgap2	9.28E-04	0.02446	1.092	7.129	7.033	8.335	8.011
10544501	Ezh2	4.59E-04	0.02404	1.091	6.658	6.459	7.707	7.592
10489831	Stau1	2.83E-04	0.02404	1.091	8.798	8.658	9.836	9.801
10385495	Cdk2ap1	2.08E-04	0.02404	1.089	7.972	8.022	9.071	9.101
10478196	Top1	7.55E-04	0.02437	1.089	8.895	8.592	9.812	9.853
10487513	Anapc1	1.50E-03	0.02748	1.089	8.451	8.071	9.426	9.274
10395103	Pxdn	4.11E-03	0.03569	1.088	8.771	9.342	10.194	10.095

10461439	Fads1	8.23E-04	0.02437	1.086	8.823	8.508	9.762	9.741
10470834	Sptan1	4.86E-04	0.02404	1.084	9.165	9.047	10.291	10.089
10394448	Pum2	2.07E-04	0.02404	1.083	7.539	7.516	8.629	8.592
10602501	Huwe1	1.76E-03	0.02788	1.083	7.994	7.637	9.020	8.778
10346523	Bzw1	2.44E-04	0.02404	1.083	8.768	8.687	9.841	9.780
10372716	Rap1b	2.98E-04	0.02404	1.083	9.564	9.428	10.550	10.608
10491595	4932438A13Rik	4.75E-04	0.02404	1.083	6.687	6.629	7.852	7.629
10440600	Cct8	5.92E-03	0.04093	1.083	8.155	7.524	8.989	8.855
10566097	Nup98	3.36E-04	0.02404	1.082	8.755	8.585	9.758	9.747
10416940	Tpm3	5.77E-04	0.02411	1.082	10.063	9.815	11.059	10.983
10524312	Ttc28	1.24E-03	0.02609	1.081	8.299	7.925	9.209	9.177
10553967	Pcsk6	2.57E-04	0.02404	1.081	8.611	8.704	9.706	9.771
10424370	Trib1	2.35E-04	0.02404	1.080	9.375	9.324	10.466	10.393
10461991	Zfand5	5.06E-04	0.02404	1.079	9.042	9.236	10.285	10.150
10539649	Ptges3	3.19E-04	0.02404	1.077	8.842	8.768	9.951	9.814
10539773	Gfpt1	9.04E-03	0.04848	1.076	7.807	7.098	8.615	8.443
10605465	Prkx	3.32E-04	0.02404	1.076	7.794	7.679	8.870	8.755
10597758	Csrnp1	4.05E-04	0.02404	1.076	9.101	8.907	10.058	10.101
10585970	Myo9a	2.66E-04	0.02404	1.075	8.205	8.170	9.317	9.207
10539861	Rpn1	2.65E-04	0.02404	1.072	8.326	8.243	9.394	9.320
10558454	Glrx3	4.61E-04	0.02404	1.072	6.304	6.304	7.484	7.267
10487277	Trpm7	8.97E-04	0.02446	1.070	6.755	7.000	8.050	7.845
10547436	Wnk1	3.22E-04	0.02404	1.069	9.763	9.613	10.766	10.748
10601192	Taf1	1.37E-03	0.02702	1.067	6.524	6.190	7.516	7.331
10403680	Arid4b	5.02E-04	0.02404	1.066	6.895	7.000	8.114	7.913
10420694	Ints6	5.23E-04	0.02411	1.065	7.426	7.651	8.633	8.574
10530633	Sgcb	1.07E-03	0.02523	1.063	7.516	7.180	8.443	8.379
10436734	Bach1	1.17E-03	0.02585	1.063	8.103	7.748	8.994	8.983
10515337	Nasp	5.43E-04	0.02411	1.063	6.066	6.109	7.267	7.033
10606600	Pcdh19	3.81E-04	0.02404	1.063	7.615	7.762	8.801	8.700

10428579	Ext1	7.09E-03	0.04347	1.062	9.788	9.122	10.524	10.511
10358607	Hmcn1	2.82E-03	0.03149	1.062	5.700	5.459	6.858	6.426
10458213	Etf1	3.27E-04	0.02404	1.061	8.077	8.129	9.234	9.095
10521205	Sh3bp2	1.17E-03	0.02581	1.061	6.322	6.000	7.150	7.295
10575160	Nfat5	1.58E-03	0.02769	1.061	7.845	8.214	9.167	9.014
10345241	Dst	9.03E-04	0.02446	1.060	6.858	6.741	8.006	7.714
10344981	Pi15	6.26E-04	0.02411	1.059	8.200	7.943	9.127	9.134
10530225	Pds5a	4.76E-04	0.02404	1.059	8.000	7.788	8.963	8.943
10565759	Uvrag	2.95E-04	0.02404	1.059	7.547	7.585	8.683	8.566
10346562	Cflar	9.86E-04	0.02494	1.058	6.820	6.555	7.839	7.651
10457429	Rock1	2.55E-03	0.03008	1.056	8.335	7.877	9.222	9.103
10355567	Tmbim1	1.76E-03	0.02783	1.056	8.785	9.197	10.035	10.059
10428763	Atad2	9.59E-04	0.02478	1.055	5.459	5.555	6.714	6.409
10437222	Hnrnpa3	3.46E-03	0.03374	1.055	9.122	8.622	10.006	9.847
10460585	Fosl1	5.45E-04	0.02411	1.055	8.155	8.379	9.292	9.351
10585214	Cryab	6.65E-04	0.02411	1.054	8.741	8.492	9.713	9.629
10520362	Insig1	3.03E-04	0.02404	1.052	8.775	8.721	9.855	9.745
10498038	Elf2	4.66E-04	0.02404	1.052	7.524	7.721	8.700	8.647
10344741	Hnrnpa3	3.87E-03	0.03507	1.051	8.934	8.414	9.801	9.649
10515113	Hnrnpa3	3.87E-03	0.03507	1.051	8.934	8.414	9.801	9.649
10603708	Cask	1.10E-03	0.02531	1.050	8.028	7.735	9.014	8.849
10358849	Dhx9	3.22E-04	0.02404	1.050	7.508	7.484	8.611	8.480
10427538	Nipbl	3.06E-04	0.02404	1.049	7.570	7.562	8.676	8.555
10453451	Calm2	6.62E-04	0.02411	1.049	10.453	10.282	11.514	11.320
10582997	Casp4	4.68E-03	0.03731	1.048	8.155	8.721	9.533	9.439
10502224	Sgms2	2.29E-03	0.02964	1.048	7.443	7.200	8.558	8.180
10545308	Kdm3a	2.98E-03	0.03178	1.047	6.883	7.349	8.243	8.082
10493664	Nup210l	2.01E-03	0.02882	1.046	5.555	5.728	6.883	6.492
10556216	Ipo7	2.62E-04	0.02404	1.044	7.435	7.508	8.516	8.516
10352514	Eprs	3.58E-03	0.03410	1.043	8.858	8.375	9.758	9.560

10591869	Herpud2	4.67E-04	0.02404	1.042	8.155	8.313	9.217	9.335
10400357	Baz1a	2.48E-04	0.02404	1.042	6.870	6.907	7.943	7.919
10603151	Gpm6b	8.19E-03	0.04636	1.042	9.522	8.861	10.314	10.152
10399540	Pqlc3	9.94E-04	0.02494	1.042	7.304	7.267	8.484	8.170
10456579	Mex3c	2.02E-03	0.02884	1.038	8.607	9.022	9.811	9.895
10460221	Chka	3.16E-04	0.02404	1.038	6.066	6.000	7.119	7.022
10590983	Panx1	4.76E-04	0.02404	1.035	6.304	6.459	7.476	7.358
10507840	Heyl	8.58E-04	0.02437	1.035	6.392	6.109	7.313	7.257
10563659	Spty2d1	3.08E-04	0.02404	1.034	7.651	7.592	8.700	8.611
10455602	Dmxl1	6.02E-04	0.02411	1.034	6.895	6.954	8.071	7.845
10515399	Plk3	5.47E-04	0.02411	1.033	7.304	7.426	8.488	8.308
10391332	Ptrf	4.18E-04	0.02404	1.032	9.562	9.392	10.515	10.505
10394538	Acaca	1.93E-03	0.02830	1.030	6.658	6.459	7.768	7.409
10544875	Scrn1	8.93E-03	0.04834	1.030	8.140	7.451	8.817	8.833
10567941	Eif3c	9.39E-04	0.02446	1.030	8.633	8.527	9.750	9.470
10500666	Ptgfrn	7.74E-04	0.02437	1.029	9.055	8.814	10.024	9.904
10537184	Cald1	2.56E-03	0.03008	1.027	9.111	8.728	10.067	9.825
10440522	Adamts1	5.10E-04	0.02404	1.025	10.482	10.311	11.473	11.370
10399379	Pgk1	5.08E-04	0.02404	1.025	7.827	7.687	8.852	8.711
10498313	Pgk1	5.08E-04	0.02404	1.025	7.827	7.687	8.852	8.711
10497051	Negr1	7.86E-03	0.04559	1.023	6.585	5.931	7.304	7.257
10369647	Ddx50	1.55E-03	0.02748	1.023	7.098	6.989	8.243	7.889
10595298	Filip1	7.48E-04	0.02437	1.022	6.190	5.931	7.077	7.087
10374934	Psme4	5.66E-04	0.02411	1.021	7.209	7.150	8.304	8.098
10473272	Zc3h15	3.54E-04	0.02404	1.021	7.592	7.658	8.700	8.592
10376534	Mprip	6.48E-04	0.02411	1.021	8.331	8.335	9.472	9.236
10428561	Rad21	1.70E-03	0.02774	1.020	8.022	8.358	9.304	9.116
10534596	Cux1	8.62E-03	0.04752	1.020	7.087	6.426	7.839	7.714
10412011	Kif2a	2.45E-03	0.02988	1.020	7.340	6.931	8.238	8.071
10408850	Nedd9	5.89E-04	0.02411	1.019	6.409	6.229	7.276	7.401

10495054	Rhoc	7.41E-04	0.02437	1.019	9.006	8.765	9.861	9.947
10533807	Cdk2ap1	3.83E-04	0.02404	1.019	7.913	8.050	8.983	9.017
10409804	Zcchc6	7.33E-04	0.02437	1.018	7.257	7.238	8.392	8.140
10439442	Pla1a	7.37E-03	0.04429	1.017	10.314	9.676	11.017	11.008
10396896	Slc39a9	3.07E-04	0.02404	1.016	7.044	7.109	8.066	8.119
10359235	Rasal2	4.80E-04	0.02404	1.015	5.907	5.883	7.000	6.820
10480381	Arhgap21	4.50E-04	0.02404	1.015	8.901	8.983	10.031	9.883
10506939	Eps15	1.24E-03	0.02609	1.014	8.823	8.504	9.723	9.633
10388018	Dhx33	7.94E-04	0.02437	1.013	6.629	6.476	7.672	7.459
10437748	Gspt1	1.63E-03	0.02774	1.013	8.765	8.405	9.647	9.549
10400413	Ralgapa1	5.39E-03	0.03959	1.013	6.895	6.426	7.839	7.508
10476106	Snord57	6.89E-04	0.02417	1.012	8.662	8.430	9.527	9.589
10404407	Foxc1	5.00E-03	0.03844	1.011	5.833	5.322	6.700	6.476
10350113	Arl8a	3.09E-04	0.02404	1.009	8.539	8.592	9.602	9.549
10473414	Ssrp1	3.40E-04	0.02404	1.009	7.267	7.209	8.290	8.205
10540472	Bhlhe40	2.85E-03	0.03154	1.008	9.704	9.292	10.607	10.405
10520950	Pdlim1	6.78E-04	0.02411	1.008	9.182	8.948	10.074	10.073
10482731	Prpf40a	6.53E-04	0.02411	1.008	7.615	7.547	8.697	8.480
10409278	Nfil3	9.46E-03	0.04967	1.007	7.983	7.304	8.618	8.683
10571958	Sh3rf1	6.73E-03	0.04263	1.007	6.966	6.358	7.700	7.637
10436487	Vgll3	6.56E-04	0.02411	1.006	8.435	8.637	9.594	9.490
10367634	Akap12	4.39E-03	0.03643	1.005	9.331	8.804	10.102	10.043
10364888	Dot1l	1.09E-03	0.02531	1.005	7.119	6.820	8.006	7.943
10546725	Pdzrn3	2.95E-03	0.03171	1.004	7.109	6.700	8.017	7.801
10437160	Ets2	2.09E-03	0.02888	1.004	7.794	8.200	9.008	8.994
10398483	Dync1h1	1.00E-03	0.02494	1.003	8.022	8.195	9.229	8.994
10419240	Ddhd1	3.49E-03	0.03389	1.003	6.375	6.845	7.679	7.547
10590801	Birc3	5.69E-04	0.02411	1.003	8.484	8.644	9.628	9.506
10396778	Mpp5	6.33E-04	0.02411	1.002	6.807	6.807	7.919	7.700
10534343	Eln	3.68E-04	0.02404	1.001	9.331	9.290	10.365	10.259

10387372	Kdm6b	4.80E-04	0.02404	1.001	10.141	9.979	11.086	11.036
10411853	Erbb2ip	1.60E-03	0.02769	1.001	8.229	8.566	9.464	9.333
10586250	Dennd4a	7.42E-03	0.04442	1.001	5.977	6.508	7.409	7.077
10414932	Trav8-1	1.29E-03	0.02653	-1.007	8.011	7.714	6.781	6.931
10552343	Gm19246	4.38E-04	0.02404	-1.019	7.150	7.285	6.150	6.248
10591535	Mir199a-1	9.41E-04	0.02446	-1.027	7.418	7.714	6.555	6.524
10351515	Rnu1b1	3.01E-03	0.03192	-1.033	8.200	8.615	7.248	7.500
10559172	Krtap5-3	5.14E-03	0.03887	-1.039	8.855	8.604	7.426	7.954
10474687	Gm25189	3.38E-04	0.02404	-1.065	8.658	8.504	7.531	7.500
10576556	4930566D17Rik	7.59E-03	0.04494	-1.065	9.145	9.820	8.366	8.468
10581643	Gm25321	3.03E-03	0.03203	-1.067	7.087	7.375	6.375	5.954
10494413	Rnu1b1	6.23E-03	0.04144	-1.077	6.781	7.384	5.883	6.129
10494421	Rnu1b1	6.23E-03	0.04144	-1.077	6.781	7.384	5.883	6.129
10500343	Rnu1b1	6.23E-03	0.04144	-1.077	6.781	7.384	5.883	6.129
10500358	Rnu1b1	6.23E-03	0.04144	-1.077	6.781	7.384	5.883	6.129
10512937	Rnu1b1	6.23E-03	0.04144	-1.077	6.781	7.384	5.883	6.129
10582985	Casp1	1.59E-03	0.02769	-1.077	6.741	7.011	5.954	5.644
10542156	Clec2d	2.04E-04	0.02404	-1.082	8.718	8.748	7.651	7.651
10569308	Krtap5-1	3.44E-04	0.02404	-1.090	8.414	8.405	7.229	7.409
10532305	4930522L14Rik	2.85E-03	0.03154	-1.102	8.205	7.983	7.229	6.755
10582899	Gm10717	4.91E-03	0.03824	-1.107	9.877	10.502	9.061	9.103
10409970	Zfp935	5.69E-04	0.02411	-1.155	7.384	7.119	6.170	6.022
10582888	Gm10719	6.58E-03	0.04225	-1.156	10.203	10.875	9.248	9.518
10579894	Hhip	4.78E-03	0.03766	-1.165	7.044	6.392	5.492	5.615
10348739	Sned1	2.86E-04	0.02404	-1.172	9.285	9.492	8.224	8.209
10369615	Srgn	2.99E-03	0.03180	-1.173	10.699	10.145	9.333	9.165
10588201	n-R5s88	1.75E-04	0.02404	-1.174	6.658	6.768	5.524	5.555
10492888	Gm25188	4.01E-03	0.03536	-1.200	7.687	7.033	6.150	6.170
10582890	Gm10719	1.42E-03	0.02727	-1.206	8.788	9.250	7.762	7.864
10455957	Gm5970	3.50E-03	0.03389	-1.220	5.883	6.508	5.044	4.907

10544596	Tmem176b	1.40E-04	0.02404	-1.222	8.969	8.937	7.781	7.679
10405916	Zfp87	1.33E-04	0.02404	-1.226	8.262	8.353	7.066	7.098
10535900	Gm24105	2.54E-03	0.03008	-1.242	7.889	8.464	7.011	6.858
10582882	Gm10722	1.59E-03	0.02769	-1.277	9.954	10.484	8.910	8.974
10598089	mt-Te	5.23E-03	0.03910	-1.287	11.366	10.598	9.723	9.667
10526654	Gm7285	2.33E-03	0.02964	-1.291	7.948	7.562	6.700	6.229
10538150	Tmem176a	1.20E-04	0.02404	-1.328	7.877	7.807	6.585	6.443
10384223	Igfbp3	3.01E-03	0.03191	-1.354	7.889	8.551	6.755	6.977
10492953	Gm25039	1.31E-03	0.02681	-1.354	8.180	8.672	7.190	6.954
10490246	Gm14326	7.56E-05	0.02404	-1.365	9.916	9.872	8.562	8.496
10582556	n-R5s102	8.37E-04	0.02437	-1.400	7.160	7.238	6.044	5.555
10582558	n-R5s102	8.37E-04	0.02437	-1.400	7.160	7.238	6.044	5.555
10582564	n-R5s102	8.37E-04	0.02437	-1.400	7.160	7.238	6.044	5.555
10582568	n-R5s102	8.37E-04	0.02437	-1.400	7.160	7.238	6.044	5.555
10582574	n-R5s102	8.37E-04	0.02437	-1.400	7.160	7.238	6.044	5.555
10582578	n-R5s102	8.37E-04	0.02437	-1.400	7.160	7.238	6.044	5.555
10423505	Cmbl	4.16E-03	0.03586	-1.443	6.600	7.426	5.555	5.585
10582896	Gm10718	2.42E-04	0.02404	-1.525	9.908	10.238	8.629	8.468
10416057	Clu	8.22E-04	0.02437	-1.528	8.170	8.679	6.781	7.011
10582916	Gm17535	7.12E-05	0.02404	-1.549	9.510	9.681	8.093	8.000
10598057	mt-Tr	1.47E-03	0.02748	-1.595	7.055	7.714	5.907	5.672
10364784	Reep6	7.98E-04	0.02437	-1.597	9.285	9.264	7.384	7.972
10450038	Angptl4	9.82E-05	0.02404	-1.604	10.496	10.762	9.000	9.050
10512063	Gm25581	4.13E-04	0.02404	-1.613	7.983	8.349	6.392	6.714
10490913	Car3	1.06E-05	0.02404	-3.430	11.166	10.863	7.401	7.768
10523359	Cxcl13	8.56E-05	0.02404	-3.981	11.077	12.001	7.375	7.741

Table S2. Genes differentially expressed more than 2.0 fold between HFD and SD in scAPs.

Probe Set ID	Gene Symbol	Raw p-value	Benjamini-Hochberg corr. P-Value	Mean Fold Change HFD/SD (Log2)	SD_SC_AP1	SD_SC_AP2	HFD_SC_AP1	HFD_SC_AP2
10527920	Cyp51	3.44E-07	0.00400	2.281	6.304	6.170	8.500	8.535
10424349	Sqle	1.93E-06	0.00518	2.222	5.858	6.340	8.384	8.257
10482762	Idi1	1.21E-06	0.00518	2.213	5.700	5.392	7.889	7.629
10375051	Hba-a2	5.03E-06	0.00651	2.207	8.313	7.801	10.057	10.471
10566326	Trim12a	1.38E-06	0.00518	2.133	4.322	4.585	6.459	6.714
10547227	Ret	7.47E-06	0.00725	2.131	6.190	6.000	8.551	7.901
10375058	Hba-a2	1.00E-05	0.00762	2.113	8.322	7.814	9.928	10.434
10566258	Hbb-bs	2.70E-05	0.01083	2.014	8.093	7.827	9.583	10.364
10403413	Idi1	3.11E-06	0.00518	1.907	5.459	5.248	7.392	7.129
10566254	Hbb-bs	4.42E-05	0.01495	1.897	8.098	7.788	9.455	10.224
10351206	Selp	2.66E-05	0.01083	1.882	6.267	6.066	8.397	7.700
10604743	Snord61	5.33E-04	0.03609	1.873	8.028	6.845	9.000	9.620
10444778	Gm25128	2.73E-06	0.00518	1.855	7.150	7.170	8.892	9.137
10537909	Rny3	9.54E-05	0.02223	1.848	9.672	10.459	11.659	12.168
10476395	Bmp2	3.57E-06	0.00519	1.836	6.443	6.728	8.459	8.384
10574438	Cdh5	2.28E-06	0.00518	1.828	6.954	7.011	8.877	8.745
10572897	Hmox1	6.50E-06	0.00689	1.811	9.478	9.864	11.539	11.424
10496359	Emcn	1.33E-05	0.00813	1.796	5.000	4.524	6.443	6.672
10583732	Ldlr	1.03E-05	0.00762	1.778	7.814	7.768	9.801	9.338
10578916	Msmo1	9.19E-06	0.00762	1.771	6.919	6.539	8.607	8.392
10563112	Snord33	6.08E-05	0.01864	1.699	8.647	8.129	9.831	10.344
10391103	Jup	6.40E-06	0.00689	1.690	8.185	8.271	10.046	9.791
10409579	Cxcl14	2.00E-05	0.00973	1.669	8.752	9.150	10.767	10.473
10412909	Fdft1	1.19E-05	0.00773	1.643	5.931	6.248	7.820	7.644

10454077	Taf4b	6.90E-05	0.02029	1.627	6.977	7.531	9.087	8.676
10412466	Hmgcs1	1.51E-05	0.00853	1.598	8.109	8.299	9.957	9.646
10496872	Eltd1	2.57E-05	0.01083	1.579	6.129	5.672	7.484	7.476
10470320	Rpl7a	8.76E-04	0.04326	1.540	6.833	5.807	7.622	8.098
10350840	Angptl1	7.59E-05	0.02103	1.538	7.622	7.200	9.180	8.718
10565813	Snord15a	2.48E-05	0.01083	1.537	4.954	5.358	6.714	6.672
10501608	Vcam1	6.97E-05	0.02029	1.532	8.304	8.778	10.260	9.886
10501586	S1pr1	1.52E-04	0.02640	1.528	6.109	5.426	7.451	7.140
10530692	Kdr	1.54E-05	0.00853	1.524	6.248	5.977	7.607	7.665
10366476	Ptprb	1.11E-05	0.00762	1.484	5.807	5.833	7.285	7.322
10504775	Col15a1	3.47E-04	0.03006	1.473	9.281	10.015	11.344	10.897
10595033	Scg3	2.18E-04	0.02777	1.467	6.129	6.883	8.028	7.919
10565962	P2ry2	1.61E-04	0.02659	1.454	6.426	7.087	8.308	8.114
10351463	Rgs5	2.19E-04	0.02777	1.441	5.129	4.755	6.066	6.700
10392221	Pecam1	1.76E-05	0.00931	1.429	6.248	6.375	7.781	7.700
10544523	Rny1	6.60E-04	0.03862	1.426	7.044	7.435	8.234	9.098
10461154	Gm24453	1.67E-04	0.02659	1.408	5.044	4.907	6.066	6.700
10555510	Pde2a	2.12E-05	0.00990	1.376	6.672	6.658	8.082	8.000
10532711	Cmklr1	2.48E-04	0.02916	1.374	7.011	7.285	8.839	8.205
10489391	Ada	1.20E-04	0.02436	1.344	7.077	7.109	8.693	8.180
10445268	Gpr116	4.08E-05	0.01487	1.330	6.833	6.600	8.087	8.006
10468992	Frmd4a	2.46E-04	0.02916	1.330	7.140	6.644	8.426	8.017
10412298	Itga1	4.62E-04	0.03319	1.328	5.672	6.426	7.451	7.304
10515427	Gm26330	2.94E-04	0.02952	1.325	5.615	5.044	6.476	6.833
10382104	Snord104	9.78E-04	0.04448	1.322	8.718	8.711	9.576	10.497
10580033	Cd97	2.02E-04	0.02711	1.322	8.066	8.257	9.765	9.202
10506571	Dhcr24	3.11E-05	0.01208	1.320	7.492	7.435	8.836	8.731
10465895	Fads2	1.30E-04	0.02453	1.310	7.721	7.375	9.033	8.683
10388488	Fam101b	3.45E-05	0.01298	1.310	5.883	6.022	7.257	7.267
10488382	Cd93	1.55E-04	0.02640	1.310	5.644	5.170	6.833	6.600

10376885	Snord49b	4.70E-05	0.01523	1.302	8.707	8.714	9.893	10.132
10376778	Mfap4	8.22E-04	0.04148	1.296	8.195	7.672	9.568	8.892
10395163	Lamb1	1.55E-04	0.02640	1.280	9.545	9.928	11.171	10.862
10582821	Gm26397	8.06E-05	0.02103	1.279	9.226	9.520	10.559	10.745
10582958	Gucy1a2	9.27E-05	0.02205	1.277	6.209	6.524	7.748	7.539
10425283	Maff	3.96E-04	0.03153	1.275	6.741	7.384	8.229	8.447
10403352	Klf6	4.49E-05	0.01495	1.270	10.438	10.304	11.675	11.607
10520362	Insig1	8.88E-05	0.02181	1.262	9.093	8.943	10.435	10.124
10579525	Plvap	4.49E-05	0.01495	1.256	6.794	6.807	8.109	8.006
10570068	Col4a2	3.62E-04	0.03009	1.255	8.989	9.566	10.665	10.399
10347218	Gm25360	1.16E-04	0.02419	1.248	7.170	7.285	8.290	8.662
10399360	Rhob	1.02E-04	0.02320	1.246	9.207	8.883	10.365	10.217
10425695	Srebf2	1.04E-04	0.02320	1.246	7.788	8.006	9.285	9.000
10555174	Lrrc32	1.16E-04	0.02419	1.241	7.788	7.468	8.969	8.768
10544837	Tril	2.56E-04	0.02921	1.236	6.931	6.615	8.229	7.788
10531931	Sparcl1	1.31E-04	0.02453	1.232	10.918	11.158	12.426	12.113
10347748	Utp14b	5.72E-05	0.01800	1.226	6.229	6.267	7.539	7.409
10351455	Rgs5	8.12E-05	0.02103	1.226	5.672	5.459	6.714	6.870
10415636	Il17d	1.24E-04	0.02451	1.219	6.392	6.524	7.845	7.508
10598626	Tspan7	1.80E-04	0.02659	1.213	7.672	7.238	8.704	8.633
10456745	Smad7	7.46E-05	0.02103	1.204	6.714	6.883	8.044	7.960
10347222	Gm23444	2.05E-04	0.02711	1.202	8.124	8.379	9.267	9.640
10347220	Gm23444	1.85E-04	0.02659	1.196	8.066	8.340	9.238	9.560
10347224	Gm23444	1.85E-04	0.02659	1.196	8.066	8.340	9.238	9.560
10379650	Gm23444	1.85E-04	0.02659	1.196	8.066	8.340	9.238	9.560
10436666	Jam2	6.77E-04	0.03886	1.194	8.371	7.700	9.320	9.140
10538459	Aqp1	2.47E-04	0.02916	1.191	7.426	7.160	8.683	8.285
10422822	Lifr	4.14E-04	0.03213	1.190	6.629	7.160	8.209	7.960
10507840	Heyl	9.19E-04	0.04361	1.190	5.644	6.150	7.366	6.807
10474700	Thbs1	5.64E-04	0.03640	1.189	9.129	9.655	10.772	10.390

10508723	Snora61	1.56E-04	0.02640	1.181	8.775	9.093	10.029	10.200
10491721	Spry1	1.16E-03	0.04789	1.171	6.895	7.585	8.592	8.229
10586168	Snord16a	2.56E-04	0.02921	1.162	8.758	8.752	10.141	9.693
10464504	Lrp5	3.05E-04	0.02952	1.162	7.451	7.870	8.945	8.700
10346634	Nop58	5.05E-04	0.03470	1.157	8.977	9.488	10.533	10.246
10592585	Sc5d	8.03E-05	0.02103	1.155	8.011	8.028	9.192	9.157
10581151	Rrad	1.33E-04	0.02453	1.153	7.358	7.615	8.596	8.683
10405179	S1pr3	1.92E-04	0.02659	1.149	8.229	8.229	9.558	9.197
10456579	Mex3c	1.21E-04	0.02436	1.144	8.864	8.845	9.887	10.111
10519855	Cacna2d1	7.73E-04	0.04033	1.141	7.883	7.229	8.669	8.725
10546586	Tmf1	2.03E-04	0.02711	1.137	7.109	6.755	8.050	8.087
10478196	Top1	3.22E-04	0.02952	1.137	8.755	8.313	9.740	9.602
10584628	Thy1	1.79E-04	0.02659	1.131	9.931	9.618	10.923	10.889
10381371	Aoc3	2.64E-04	0.02945	1.130	8.954	9.238	10.374	10.079
10454651	Gm23639	4.39E-04	0.03255	1.128	6.672	6.907	7.687	8.150
10423109	Adamts12	2.14E-04	0.02770	1.125	9.893	10.159	11.265	11.037
10546706	Rybp	1.27E-04	0.02453	1.120	7.577	7.735	8.731	8.820
10581266	Tppp3	2.70E-04	0.02952	1.118	9.260	8.937	10.102	10.331
10554789	Ctsc	6.32E-04	0.03796	1.117	6.524	5.954	7.418	7.295
10381898	Mrc2	1.36E-04	0.02470	1.116	8.855	8.714	9.972	9.830
10381514	Cd300lg	5.59E-04	0.03640	1.114	6.700	7.238	8.145	8.022
10509280	Hspg2	3.16E-04	0.02952	1.112	9.841	10.134	11.251	10.948
10529264	Spon2	1.66E-04	0.02659	1.111	8.690	8.447	9.718	9.642
10476740	Slc24a3	4.48E-04	0.03266	1.101	6.658	7.119	8.066	7.913
10540072	Ppp1r2	2.63E-04	0.02945	1.097	8.180	7.983	9.031	9.326
10471486	Eng	2.13E-04	0.02770	1.096	8.344	8.061	9.344	9.253
10463211	Pi4k2a	2.93E-04	0.02952	1.089	7.827	7.555	8.904	8.655
10461439	Fads1	1.38E-04	0.02470	1.087	8.322	8.349	9.476	9.369
10508392	Rnf19b	1.76E-04	0.02659	1.083	7.960	8.028	9.177	8.977
10350992	Rc3h1	3.19E-04	0.02952	1.083	8.508	8.353	9.687	9.340

10536294	Peg10	3.08E-04	0.02952	1.078	8.464	8.581	9.771	9.428
10565802	Rps3	2.35E-04	0.02879	1.068	9.433	9.474	10.387	10.655
10467003	Ppp1r2	3.17E-04	0.02952	1.067	8.581	8.430	9.414	9.733
10576973	Col4a1	7.35E-04	0.04008	1.064	9.058	9.566	10.472	10.281
10568529	Ikzf5	1.01E-03	0.04470	1.057	7.426	6.883	8.340	8.082
10375002	Cpeb4	9.77E-04	0.04448	1.052	7.775	7.577	9.006	8.451
10490826	Zbtb10	2.46E-04	0.02916	1.050	7.248	7.492	8.439	8.401
10428398	Eif3e	3.04E-04	0.02952	1.050	8.687	8.527	9.524	9.790
10545588	Hk2	3.00E-04	0.02952	1.048	8.748	9.000	10.007	9.838
10381458	Rnu2-10	3.24E-04	0.02952	1.046	8.414	8.711	9.667	9.549
10584288	Robo4	1.92E-04	0.02659	1.045	5.907	5.781	6.907	6.870
10392522	Abca8a	5.96E-04	0.03714	1.043	9.834	9.653	11.000	10.573
10594636	Ppp1r2	3.47E-04	0.03006	1.042	8.180	8.071	9.011	9.324
10470318	Gm24134	3.00E-04	0.02952	1.042	9.992	9.838	10.833	11.080
10504123	4933409K07Rik	1.78E-04	0.02659	1.042	7.592	7.531	8.600	8.607
10504125	4933409K07Rik	1.78E-04	0.02659	1.042	7.592	7.531	8.600	8.607
10498935	Gucy1b3	2.00E-04	0.02711	1.040	5.672	5.672	6.781	6.644
10555179	Prkrir	3.80E-04	0.03120	1.040	7.577	7.229	8.422	8.464
10381460	Rnu2-10	3.53E-04	0.03006	1.036	8.392	8.687	9.646	9.506
10381470	Rnu2-10	3.53E-04	0.03006	1.036	8.392	8.687	9.646	9.506
10381472	Rnu2-10	3.53E-04	0.03006	1.036	8.392	8.687	9.646	9.506
10391488	Rnu2-10	3.53E-04	0.03006	1.036	8.392	8.687	9.646	9.506
10552311	Gm15470	5.77E-04	0.03671	1.031	6.644	6.304	7.644	7.366
10578688	Rnu2-10	3.50E-04	0.03006	1.027	8.371	8.644	9.604	9.466
10606600	Pcdh19	2.29E-04	0.02864	1.026	7.401	7.555	8.512	8.496
10363706	Jmjd1c	8.97E-04	0.04337	1.021	8.687	8.468	9.836	9.360
10440522	Adamts1	2.34E-04	0.02879	1.019	10.407	10.548	11.497	11.495
10492195	Tsc22d2	9.85E-04	0.04448	1.017	8.392	8.778	9.791	9.414
10487252	Gabpb1	2.65E-04	0.02945	1.017	6.524	6.700	7.665	7.592
10495993	Elovl6	1.06E-03	0.04612	1.014	7.814	7.267	8.592	8.516

10553967	Pcsk6	4.94E-04	0.03452	1.014	8.928	9.063	9.836	10.182
10349947	Fmod	6.60E-04	0.03862	1.013	6.700	7.119	7.994	7.852
10412038	Zswim6	3.90E-04	0.03137	1.011	7.190	6.943	8.165	7.989
10441509	Ppp1r2	3.27E-04	0.02952	1.005	8.257	8.248	9.140	9.375
10535807	Flt1	6.19E-04	0.03751	1.000	5.807	6.150	7.087	6.870
10455954	Gm4951	1.26E-03	0.04986	-1.037	8.234	8.836	7.577	7.418
10450344	C2	7.63E-04	0.04008	-1.041	9.823	9.620	8.443	8.919
10598087	ND6	1.63E-04	0.02659	-1.057	10.846	10.768	9.735	9.765
10598976	Timp1	1.90E-04	0.02659	-1.097	8.983	8.745	7.707	7.827
10582896	Gm10718	1.10E-04	0.02419	-1.231	10.391	10.474	9.031	9.373
10538150	Tmem176a	8.99E-05	0.02181	-1.236	7.615	7.919	6.555	6.508
10542156	Clec2d	6.70E-04	0.03862	-1.243	9.574	8.922	7.820	8.190
10408200	Hist1h4f	1.11E-03	0.04696	-1.274	7.562	7.547	5.833	6.728
10425049	Apol9a	8.30E-05	0.02103	-1.299	7.768	7.577	6.209	6.539
10378855	Ssh2	1.14E-04	0.02419	-1.325	6.755	6.409	5.087	5.426
10523359	Cxcl13	2.54E-04	0.02921	-1.332	12.294	11.891	10.504	11.016
10345791	Il1rl1	3.83E-04	0.03120	-1.360	7.219	6.629	5.322	5.807
10412126	Il31ra	3.66E-04	0.03022	-1.365	7.484	6.728	5.700	5.781
10598089	mt-Te	1.10E-05	0.00762	-1.596	11.227	10.966	9.441	9.560
10494643	Hmgcs2	1.93E-05	0.00973	-1.663	8.592	9.014	7.022	7.257
10490913	Car3	7.55E-04	0.04008	-1.688	10.713	11.152	8.662	9.827
10513504	Mup2	1.13E-03	0.04726	-1.714	7.229	5.931	4.644	5.087
10455957	Gm5970	3.15E-04	0.02952	-1.742	6.755	7.833	5.459	5.644
10513428	Mup2	7.09E-04	0.03931	-1.794	7.119	5.883	4.459	4.954
10513472	Mup10	3.98E-04	0.03153	-1.802	7.340	6.229	4.755	5.209
10513437	Mup10	4.02E-04	0.03158	-1.936	7.219	6.044	4.392	5.000
10513455	Mup2	4.69E-04	0.03329	-1.976	7.392	6.190	4.459	5.170
10513420	Mup2	2.98E-04	0.02952	-2.066	7.570	6.358	4.585	5.209

Table S3. GO analysis of viAP signature gene list.

Term	Count	%	PValue	Genes
GO:0007049~cell cycle	38	0,99	2,64E-09	GAS2L3, PRC1, NEDD9, AHCTF1, ANLN, ITGB1, STARD13, RAD21, NIPBL, SKIL, CCNA2, ANAPC1, CKAP2, TAF1, KIF11, MKI67, PDS5A, PDPN, DLGAP5, NASP, NUF2, TPX2, RACGAP1, SMC2, SMC4, CCNB1, MAD2L1, CCNB2, PLK3, MAPK6, PLK1, PTP4A1, CDK2AP1, ARL8A, STMN1, DST, CALM2, MYH10
GO:0022402~cell cycle process	27	0,70	1,31E-07	GAS2L3, ANAPC1, KIF11, PDS5A, MKI67, DLGAP5, NUF2, TPX2, NEDD9, AHCTF1, ANLN, RACGAP1, ITGB1, SMC2, SMC4, CCNB1, RAD21, MAD2L1, CCNB2, NIPBL, PLK1, ARL8A, STMN1, SKIL, CCNA2, DST, MYH10
GO:0000087~M phase of mitotic cell cycle	18	0,47	4,67E-07	ANAPC1, KIF11, PDS5A, DLGAP5, NUF2, AHCTF1, NEDD9, ANLN, SMC2, SMC4, CCNB1, NIPBL, MAD2L1, CCNB2, RAD21, PLK1, ARL8A, CCNA2
GO:0000278~mitotic cell cycle	20	0,52	6,12E-07	ANAPC1, KIF11, PDS5A, DLGAP5, NUF2, AHCTF1, NEDD9, ANLN, ITGB1, SMC2, SMC4, CCNB1, RAD21, NIPBL, MAD2L1, CCNB2, PLK1, ARL8A, STMN1, CCNA2
GO:0051301~cell division	21	0,55	1,27E-06	ANAPC1, KIF11, PRC1, PDS5A, ROCK1, NUF2, AHCTF1, NEDD9, ANLN, RACGAP1, SMC2, SMC4, CCNB1, RAD21, MAD2L1, CCNB2, PLK1, ARL8A, CCNA2, TOP2A, MYH10
GO:0000279~M phase	21	0,55	1,43E-06	ANAPC1, KIF11, MKI67, PDS5A, DLGAP5, NUF2, TPX2, AHCTF1, NEDD9, ANLN, SMC2, SMC4, CCNB1, RAD21, NIPBL, MAD2L1, CCNB2, PLK1, ARL8A, STMN1, CCNA2
GO:0000280~nuclear division	17	0,44	1,73E-06	ANAPC1, KIF11, PDS5A, NUF2, AHCTF1, NEDD9, ANLN, SMC2, SMC4, CCNB1, NIPBL, MAD2L1, CCNB2, RAD21, PLK1, ARL8A, CCNA2
GO:0007067~mitosis	17	0,44	1,73E-06	ANAPC1, KIF11, PDS5A, NUF2, AHCTF1, NEDD9, ANLN, SMC2, SMC4, CCNB1, NIPBL, MAD2L1, CCNB2, RAD21, PLK1, ARL8A, CCNA2
GO:0048285~organelle fission	17	0,44	2,79E-06	ANAPC1, KIF11, PDS5A, NUF2, AHCTF1, NEDD9, ANLN, SMC2, SMC4, CCNB1, NIPBL, MAD2L1, CCNB2, RAD21, PLK1, ARL8A, CCNA2
GO:0022403~cell cycle phase	22	0,57	3,74E-06	ANAPC1, KIF11, MKI67, PDS5A, DLGAP5, NUF2, TPX2, AHCTF1, NEDD9, ANLN, ITGB1, SMC2, SMC4, CCNB1, RAD21, NIPBL, MAD2L1, CCNB2, PLK1, ARL8A, STMN1, CCNA2
GO:0007010~cytoskeleton organization	19	0,50	1,27E-04	KIF11, ROCK1, TRPM7, CEP192, ELN, NUF2, TPX2, ACTN1, RACGAP1, DAAM1, ITGB1, FLNA, YWHAH, FAT1, STMN1, DST, DIAP3, SGCB, MYH10
GO:0000910~cytokinesis	6	0,16	1,90E-04	ROCK1, PRC1, AHCTF1, ANLN, RACGAP1, MYH10
GO:0051270~regulation of cell motion	10	0,26	3,35E-04	LAMA2, GNA13, LAMA4, PDPN, CLIC4, RRAS2, PTP4A1, TMOD3, STMN1, VCL

Table S4. GO analysis of scAP signature gene list.

Term	Count	%	PValue	Genes
GO:0007155~cell adhesion	14	0,52	9,35E-07	SELP, EMCN, ITGA1, HSPG2, COL15A1, CDH5, JUP, CD97, CD93, PECAM1, THBS1, SPON2, ENG, AOC3
GO:0022610~biological adhesion	14	0,52	9,54E-07	SELP, EMCN, ITGA1, HSPG2, COL15A1, CDH5, JUP, CD97, CD93, PECAM1, THBS1, SPON2, ENG, AOC3
GO:0030334~regulation of cell migration	6	0,22	5,62E-05	SELP, S1PR1, PECAM1, ROBO4, THBS1, ADA
GO:0051270~regulation of cell motion	6	0,22	1,16E-04	SELP, S1PR1, PECAM1, ROBO4, THBS1, ADA
GO:0040012~regulation of locomotion	6	0,22	1,32E-04	SELP, S1PR1, PECAM1, ROBO4, THBS1, ADA
GO:0001525~angiogenesis	6	0,22	3,19E-04	EMCN, FLT1, S1PR1, ROBO4, ENG, KDR
GO:0016126~sterol biosynthetic process	4	0,15	3,21E-04	SC5D, HMGCS2, DHCR24, FDFT1
GO:0016125~sterol metabolic process	5	0,19	3,89E-04	SC5D, HMGCS2, SREBF2, DHCR24, FDFT1
GO:0002685~regulation of leukocyte migration	3	0,11	5,46E-04	SELP, THBS1, ADA
GO:0001568~blood vessel development	7	0,26	7,67E-04	EMCN, FLT1, S1PR1, ROBO4, ENG, CDH5, KDR
GO:0001944~vasculature development	7	0,26	8,70E-04	EMCN, FLT1, S1PR1, ROBO4, ENG, CDH5, KDR
GO:0009611~response to wounding	8	0,30	8,90E-04	KLF6, SELP, S1PR3, IL17D, BMP2, PECAM1, C2, THBS1

Table S5. GSEA analysis of AP fractions, related to Figure 1D. Analyzed gene sets include Gene Onthology, KEGG and Reactome-derived cell cycle pathways as well as custom-curated genesets from a 3T3-L1 differentiation time course.

Table S5. GSEA analysis of viAP or scAP gene sets, including GO, KEGG, Reactome and 3T3-L1 proliferation.

(i) Gene Onthology - Cell cycle process (GO:0022402)

Gene Symbol	Rank in Gene List	Rank Metric Score	Running ES	Core Enrichment
ANLN	2	3.1543715	0.0294066	Yes
PRC1	7	2.524915934	0.05274887	Yes
RACGAP1	9	2.460883379	0.07573627	Yes
KPNA2	18	2.279769421	0.09645328	Yes
KIF2C	19	2.273268223	0.11776369	Yes
KIF11	28	2.165422916	0.13740878	Yes
KIF23	29	2.164519072	0.15769975	Yes
CCNA2	40	2.050919771	0.17610785	Yes
CENPE	49	2.009554863	0.19429179	Yes
MAD2L1	64	1.90438962	0.2109991	Yes
PAM	67	1.895977497	0.22860907	Yes
STMN1	89	1.744995475	0.24324962	Yes
NUSAP1	96	1.718858957	0.25887203	Yes
KIF15	98	1.711407304	0.2748336	Yes
PLK1	125	1.612931132	0.28782716	Yes
BUB1	129	1.603421807	0.3026128	Yes
KNTC1	135	1.580591321	0.31702086	Yes
CENPF	179	1.445029497	0.32704997	Yes
SMC4	204	1.410922408	0.33831343	Yes
NCAPH	209	1.403774023	0.35114574	Yes
MYH10	223	1.383441329	0.3630513	Yes
TPX2	256	1.318456888	0.37279359	Yes
CDK2	278	1.294804931	0.3832139	Yes
TAF1	303	1.261969686	0.393081	Yes
POLA1	323	1.2384547	0.40313667	Yes
BIRC5	341	1.2190485	0.41317397	Yes
CDKN3	359	1.197926521	0.42301327	Yes
CKAP5	396	1.16419065	0.43098226	Yes
CDKN2C	406	1.155764937	0.44108066	Yes
MRE11A	422	1.143801093	0.45057616	Yes
ANAPC4	432	1.13710022	0.4604996	Yes
POLE	479	1.102212071	0.46706966	Yes
DBF4	520	1.075716972	0.47388208	Yes
ATM	530	1.072071791	0.48319593	Yes
DCTN2	596	1.038625002	0.48761582	Yes

NDE1	619	1.028540373	0.49545828	Yes
KIF22	625	1.022356749	0.50463325	Yes
CDK2AP1	657	0.997994363	0.5114532	Yes
BUB1B	667	0.993213177	0.5200278	Yes
CUL3	700	0.983086407	0.5266262	Yes
GSPT1	759	0.951378286	0.5308008	Yes
NUMA1	791	0.931761265	0.5369999	Yes
PIM2	983	0.851073742	0.5293557	Yes
FBXO5	1055	0.821902871	0.5312532	Yes
RB1	1184	0.777927816	0.5280763	Yes
CHEK1	1279	0.742491186	0.5273481	Yes
NOLC1	1283	0.741458833	0.53405344	Yes
RAD21	1320	0.730436504	0.53795624	Yes
SKP2	1346	0.72247225	0.54268414	Yes
LIG3	1439	0.696337879	0.5416869	Yes
BRCA2	1536	0.672894418	0.5401427	Yes
APBB2	1655	0.641502857	0.53650486	Yes
AURKA	1709	0.629060864	0.53806686	Yes
TARDBP	1718	0.625991106	0.5432808	Yes
LATS2	1722	0.625488341	0.54889894	Yes
SASS6	1788	0.611242712	0.5493124	Yes
RAD54L	1809	0.606428683	0.5533614	Yes
LATS1	1814	0.6059798	0.5587149	Yes
PPP6C	1819	0.604872465	0.564058	Yes
TGFB1	1836	0.602302194	0.56839556	Yes
CUL4A	1855	0.598160505	0.5725306	Yes
SUGT1	1859	0.597976267	0.5778909	Yes
CIT	1939	0.582791448	0.57689255	Yes
ZWINT	1993	0.569817364	0.5778992	Yes
CUL5	1996	0.569094479	0.5830705	Yes
MPHOSPH6	2020	0.562837064	0.5864655	Yes
CDK4	2079	0.552308619	0.58689904	Yes
SSSCA1	2089	0.551311255	0.59133106	Yes
KATNA1	2391	0.493618101	0.5713388	No
CDKN2D	2539	0.467904627	0.5637015	No
BRSK1	2596	0.458308399	0.56341743	No
PAFAH1B1	2649	0.450781614	0.56339	No
TTK	2676	0.444807529	0.5654332	No
NEK2	2786	0.430341423	0.56055194	No
CDC25C	3011	0.393791139	0.54592186	No
AKAP8	3134	0.372583807	0.53943586	No
RAD51	3262	0.35540098	0.5323798	No
XRCC2	3291	0.350657016	0.53337675	No
PKMYT1	3401	0.333991289	0.5275923	No
CDC6	3447	0.327204347	0.52697897	No
ACVR1B	3617	0.306631088	0.51603043	No
CUL2	3825	0.282477021	0.5017473	No

PML	3833	0.280351311	0.5038029	No
RAN	4097	0.244674951	0.48458502	No
KHDRBS1	4208	0.232700199	0.47776923	No
CDC23	4374	0.214097604	0.4662804	No
ESPL1	4732	0.172912002	0.43870127	No
CDC27	4846	0.161003545	0.430968	No
CEP250	4887	0.15591839	0.4291579	No
ABL1	4900	0.154215306	0.42962205	No
BCAT1	4975	0.146133363	0.4249393	No
MAD2L2	5151	0.128393337	0.41182914	No
ZW10	5216	0.122327916	0.40774113	No
PRMT5	5311	0.112531833	0.40110752	No
CDKN2A	5540	0.091553234	0.383317	No
INHBA	5658	0.08187785	0.37451476	No
APBB1	5721	0.073735677	0.37013483	No
CDC25B	5785	0.066227935	0.36560273	No
DDX11	5836	0.06057601	0.36208093	No
E2F1	5921	0.051422402	0.3556924	No
CCNA1	6164	0.027398486	0.33615535	No
STAG3	6247	0.01876853	0.32962427	No
DCTN3	6286	0.013902129	0.32664648	No
CDKN1A	6296	0.012587654	0.32602832	No
RAD1	6379	0.005243086	0.31937048	No
ANAPC11	6618	-0.017831329	0.3000709	No
EREG	6688	-0.023937397	0.2946516	No
CNTROB	6715	-0.027771153	0.29278532	No
RINT1	6749	-0.030550392	0.29037255	No
CDK10	6776	-0.032512147	0.2885507	No
UBE2C	6813	-0.035321131	0.28593728	No
CDC7	6898	-0.044279102	0.27948177	No
TPD52L1	6935	-0.04766335	0.27698404	No
PCBP4	7041	-0.058127802	0.2689407	No
CD28	7044	-0.058268771	0.26932332	No
MAP3K11	7068	-0.060343318	0.26800779	No
SMC3	7136	-0.066774957	0.2631536	No
GFI1	7203	-0.073758021	0.25844672	No
CDKN2B	7561	-0.109492771	0.23027308	No
RAD17	7578	-0.110995069	0.2300049	No
TBRG4	7625	-0.115101203	0.22732143	No
CHFR	7654	-0.117540561	0.2261331	No
SAC3D1	7738	-0.128427342	0.2205482	No
PPP5C	7816	-0.136707246	0.21553169	No
NEK6	8171	-0.172889337	0.18819773	No
CDCA5	8202	-0.17490384	0.18738355	No
CDC16	8226	-0.176530629	0.18715717	No
POLD1	8413	-0.195779204	0.173779	No
MDM4	8555	-0.209196404	0.16420728	No

TUBE1	8595	-0.21297738	0.16301388	No
MPHOSPH9	8654	-0.218621448	0.16031933	No
HSPA2	8694	-0.223595589	0.15922546	No
CUL1	8734	-0.227959901	0.15817252	No
TOP3A	8746	-0.229705051	0.15942614	No
NPM1	8833	-0.240442485	0.15464593	No
TGFA	8992	-0.259346068	0.14415386	No
TRIAP1	9121	-0.27336973	0.13624702	No
SMC1A	9177	-0.278600484	0.13436012	No
EGF	9238	-0.28523013	0.13212639	No
RAD50	9301	-0.292402506	0.12979631	No
GFI1B	9337	-0.295924187	0.12970766	No
USH1C	9417	-0.306468666	0.12611896	No
CETN1	9482	-0.313052088	0.12381888	No
NBN	9558	-0.320789099	0.1206916	No
RAD54B	9569	-0.322154075	0.12289365	No
CDK6	9581	-0.323848218	0.1250298	No
RAD52	9597	-0.325732499	0.12685643	No
RCC1	9671	-0.336087912	0.12403616	No
MSH5	9827	-0.359057128	0.11472419	No
TUBG1	9998	-0.381739229	0.10439795	No
TIPIN	10316	-0.425218672	0.08245575	No
TIMELESS	10377	-0.434457988	0.08162094	No
CETN3	10550	-0.458792418	0.07185344	No
DUSP13	10578	-0.464209557	0.07399669	No
ANAPC5	10766	-0.498103142	0.06337082	No
ACVR1	10865	-0.518298447	0.06021382	No
GML	10915	-0.527682364	0.06115265	No
CDKN1C	11104	-0.568807721	0.05110779	No
KRT7	11226	-0.596852481	0.04680595	No
NPM2	11285	-0.608468413	0.04776596	No
CDKN1B	11353	-0.624690235	0.0481419	No
DMC1	11473	-0.663129747	0.04462495	No
PIN1	11494	-0.668754756	0.04925824	No
ANAPC10	11717	-0.753852069	0.03816709	No
PTPRC	12318	-1.791885972	0.00588911	No

Table S6. Sequences of oligonucleotide primers.

Gene symbol	5' Oligonucleotide	3' Oligonucleotide
Adipoq	CGTGATGGCAGAGATGGCACTC	CCTTAGGACCAAGAAGACCTGCATC
aP2 (Fabp4)	TTCGATGAAATCACCGCAGA	AGGGCCCCGCCATCT
Cd68	TGGCGCAGAATTCATCTCTTC	GGTCAAGGTGAACAGCTGGAG
Cebpa	GAACAGCTGAGCCGTGAACT	AAACCATCCTCTGGGTCTCC
HO-1 (Hmox1)	TCCGCATACAACCAGTGAGTG	GCTAGTTCAGGCTGGTTCTGC
hLPL	TGGAGGTACTTTTCAGCCAGGAT	TCGTGGGAGCACTCACTAGCT
Pdgfra	AGCAGGCAGGGCTTCAACGG	ACACAGTCTGGCGTGCGTCC
Pparg	GCATGGTGCCTTCGCTGA	TGGCATCTCTGTGTCAACCATG
hRBP4	GCCTCTTTCTGCAGGACAAC	GAAGGTGCCCACCATGTCT

hRPLP0	GTCATCCAGCAGGTGTTCGAC	CTCCAGGAAGCGAGAATGCAG
Rplp0	GCCAATAAGGTGCCAGCTGCTG	GAAGGAGGTCTTCTCGGGTCCTAG

Aliases of gene symbols given in parenthese; h corresponds to human genes.
